# Supplementary material for: Aggregated Genomic Data as Cohort-Specific Allelic Frequencies can Boost Variants and Genes Prioritization in Non-Solved Cases of Inherited Retinal Dystrophies
Source: Int J Mol Sci. 2022 Jul 29;23(15):8431. doi: 10.3390/ijms23158431 (PMC9368980; doi:10.3390/ijms23158431)
Supplement: Supplementary file 1 [file ijms-23-08431-s001.zip › ijms-1808225-supplementary.pdf]

Supplementary Material: Supplementary Tables S1-12; Supplementary Figures S1-6.

Aggregated genomic data as cohort-specific allelic frequencies can boost variants and genes prioritization in non-solved cases of inherited retinal dystrophies

Ionut-Florin Iancu[1,2], Irene Perea-Romero[1,2], Gonzalo Núñez-Moreno[1,2,3], Lorena de la Fuente[1,3], Raquel Romero[1,2], Almudena Ávila-Fernandez[1,2], María José Trujillo-Tiebas[1,2], Rosa Riveiro-Álvarez[1,2], Berta Almoguera[1,2], Inmaculada Martín-Mérida[1,2], Marta Del Pozo-Valero[1,2], Alejandra Damián-Verde[1], Marta Cortón[1,2], Carmen Ayuso[1,2]# and Pablo Minguez[1,2,3]#

[1]Department of Genetics, Health Research Institute–Fundación Jiménez Díaz University Hospital, Universidad Autónoma de Madrid (IIS-FJD, UAM), Madrid, Spain.

[2]Center for Biomedical Network Research on Rare Diseases (CIBERER), ISCIII, Madrid, Spain.

[3]Bioinformatics Unit, Health Research Institute–Fundación Jiménez Díaz University Hospital, Universidad Autónoma de Madrid (IIS-FJD, UAM), Madrid, Spain.

# Corresponding authors: cayuso@fjd.es and pablo.minguez@quironsalud.es

Supplementary Tables

Table S1. Number of deleterious and benign within the prioritized (IRD-MFV) and non-prioritized sets in IRD solved cases, including those in all genes and in genes from the inherited retinal dystrophies (IRD) panel, the other eye related diseases (OERD) and non-eye related diseases (NRD). P-values of the Fishers' exact test are shown.

	GENE_PANEL	Clinical Significance	IRD-MFV	Non-prioritized	p-value
SOLVED	ALL genes	Deleterious	404	4191	4.77E-56
		Benign	762	23020	
	IRD genes	Deleterious	177	593	5.97E-61
		Benign	49	1926	
	OERD genes	Deleterious	80	1457	1.71E-06
		Benign	314	10843	
	NRD genes	Deleterious	147	2141	2.75E-08
		Benign	399	10251	

Table S2. Number of deleterious and benign within the prioritized (IRD-MFV) and non-prioritized sets in IRD non-solved cases, including those in all genes and in genes from the inherited retinal dystrophies (IRD) panel, the other eye related diseases (OERD) and non-eye related diseases (NRD). P—values of the Fishers' exact test are shown.

	GENE_PANEL	Clinical Significance	IRD-MFV	Non-prioritized	p-value
NON-SOLVED	ALL genes	Deleterious	148	2143	1.69E-32
		Benign	399	21222	
	IRD genes	Deleterious	7	193	6.6E-02
		Benign	31	1844	
	OERD genes	Deleterious	37	770	3.25E-06
		Benign	186	9805	
	NRD genes	Deleterious	104	1180	1.90E-28
		Benign	182	9573	

Table S3. Number of deleterious and benign within the prioritized (IRD-MFV) and non-prioritized sets in IRD solved syndromic (SY), IRD non-syndromic (NSY) and macular dystrophies (MD) cases, including those in all genes and in genes from the inherited retinal dystrophies (IRD) panel, the other eye related diseases (OERD) and non-eye related diseases (NRD). P-values of the Fishers' exact test are shown.

	GENE_PANEL	IRD type	Clinical Significance	IRD-MFV	Non-prioritized	p-value
SOLVED	IRD genes	SY	Benign	11	427	7.84E-06
			Deleterious	13	68	
		NSY	Benign	23	585	8.09E-26
			Deleterious	86	214	
		MD	Benign	18	1099	2.19E-25
			Deleterious	56	239	
	OERD genes	SY	Benign	52	4184	2.29E-08
			Deleterious	19	257	
		NSY	Benign	207	7094	2.28E-06
			Deleterious	52	801	
		MD	Benign	153	6076	1.35E-04
			Deleterious	34	618	
	NRD genes	SY	Benign	68	4126	1.89E-12
			Deleterious	36	414	
		NSY	Benign	228	6795	1.67E-13
			Deleterious	103	1176	
		MD	Benign	200	5897	3.02E-03
			Deleterious	48	871	

Table S4. Number of deleterious and benign within the prioritized (IRD-MFV) and non-prioritized sets in IRD non-solved syndromic (SY), non-syndromic (NSY) and macular dystrophies (MD) cases, including those in all genes and in genes from the inherited retinal dystrophies (IRD) panel, the other eye related diseases (OERD) and non-eye related diseases (NRD). P-values of the Fishers' exact test are shown.

	GENE_PANEL	IRD type	Clinical Significance	IRD-MFV	Non-prioritized	p-value
NON-SOLVED	IRD genes	SY	Benign	7	355	1.39E-06
			Deleterious	7	14	
		NSY	Benign	16	494	3.71E-04
			Deleterious	8	39	
		MD	Benign	29	935	1.03E-01
			Deleterious	5	73	
	OERD genes	SY	Benign	57	3297	9.57E-18
			Deleterious	25	100	
		NSY	Benign	145	5742	1.42E-06
			Deleterious	31	415	
		MD	Benign	118	5176	7.03E-10
			Deleterious	31	311	
	NRD genes	SY	Benign	29	3350	2.69E-26
			Deleterious	35	194	
		NSY	Benign	129	6795	7.84E-23
			Deleterious	76	1176	
		MD	Benign	105	5186	4.57E-18
			Deleterious	54	499	

Table S5. Genes that ACMG recommends to report secondary findings. These genes are filtered out from the OERD and NRD gene panels.

Gene			
ACTA2	HFE	PCSK9	TGFBR2
ACTC1	HNF1A	PKP2	TMEM127
ACVRL1	KCNH2	PMS2	TMEM43
APC	KCNQ1	PRKAG2	TNNI3
APOB	LDLR	PTEN	TNNT2
ATP7B	LMNA	RB1	TP53
BMPR1A	MAX	RET	TPM1
BRCA1	MEN1	RPE65	TRDN
BRCA2	MLH1	RYR1	TSC1
BTD	MSH2	RYR2	TSC2
CACNA1S	MSH6	SCN5A	TTN
CASQ2	MUTYH	SDHAF2	VHL
COL3A1	MYBPC3	SDHB	WT1
DSC2	MYH11	SDHC	
DSP	MYH7	SDHD	
ENG	MYL2	SMAD3	
FBN1	MYL3	SMAD4	
FLNC	NF2	STK11	
GAA	OTC	TGFBR1	
GLA	PALB2	TGFBR2	

Table S6. Genes prioritized in solved cases of inherited retinal dystrophies. They are classified in three gene panels: genes from the inherited retinal dystrophies (IRD) panel, other eye related diseases (OERD) and non-eye related diseases (NRD).

Gene	Deleterious	Benign	FDR	Gene Panel
ABCA4	84	24	1.13E-08	IRD
USH2A	73	76	7.35E-11	IRD
MYO7A	31	41	3.94E-06	IRD
EYS	20	11	3.20E-03	IRD
ADGRV1	14	67	6.73E-04	IRD
CRB1	13	7	4.81E-02	IRD
PROM1	13	7	1.59E-02	IRD
CNGB3	13	8	5.10E-03	IRD
VPS13B	13	37	1.59E-02	IRD
CEP290	12	23	5.67E-04	IRD
RPGR	10	7	1.62E-02	IRD
CDH23	9	35	1.77E-03	IRD
DYNC2H1	9	39	7.28E-04	IRD
TRPM1	9	14	1.92E-02	IRD

AHI1	9	29	2.41E-03	IRD
OPA1	8	19	2.74E-03	IRD
HMCN1	7	45	1.26E-02	IRD
COL11A1	6	36	1.47E-02	IRD
PRPF31	6	5	2.07E-02	IRD
BBS9	6	9	1.52E-02	IRD
RPE65	5	5	2.89E-02	IRD
GRM6	5	14	2.24E-02	IRD
NEB	22	84	1.23E-05	OERD
PAH	18	8	1.38E-02	OERD
DNAH11	14	36	1.70E-04	OERD
DNAH5	13	47	1.22E-02	OERD
ATM	12	60	1.22E-02	OERD
HSPG2	11	53	9.11E-04	OERD
LAMA2	9	49	1.75E-03	OERD
PLEC	9	87	1.62E-02	OERD
MPDZ	9	28	6.33E-03	OERD
ABCC8	8	9	1.26E-02	OERD
COL5A2	8	36	4.81E-02	OERD
COL7A1	8	26	3.67E-02	OERD
C1QTNF5	7	8	1.59E-02	OERD
DNAH9	7	6	1.44E-02	OERD
FLNB	7	37	1.78E-02	OERD
DOCK8	7	28	1.62E-02	OERD
CENPJ	6	8	2.89E-02	OERD
FANCI	6	24	3.19E-02	OERD
GALK1	6	9	1.28E-02	OERD
XYLT1	5	11	1.42E-02	OERD
ABCA12	5	15	4.04E-02	OERD
COL4A3	5	15	2.31E-02	OERD
MME	5	5	4.57E-02	OERD
EFHC1	5	14	1.52E-02	OERD
OBSCN	16	95	1.62E-02	NRD
DYSF	9	43	2.74E-03	NRD
SPTBN5	8	8	3.05E-02	NRD
OTOF	8	24	6.49E-03	NRD

SPTB	6	24	4.04E-02	NRD
OBSL1	6	30	1.59E-02	NRD
ANO3	5	13	1.59E-02	NRD
ZFHX3	5	10	2.89E-02	NRD
MYO1C	5	16	2.37E-02	NRD
CLCN1	5	21	2.24E-02	NRD

Table S7. Diseases included in the allele frequency database, and number (N) of cases of each disease. Diseases are classified in three categories: i) Inherited Retinal Dystrophies (IRD), ii) other eye related diseases (OERD) and iii) non-related diseases (NRD).

Disease	N	Group
Inherited retinal dystrophies	1766	IRD
Mixed conditions	1648	NRD
Encephalopathies-ID-Epilepsy	735	NRD
Cardiopathy	325	NRD
Peripheral neuropathies	209	NRD
Optic atrophy	200	OERD
Hearing loss	149	NRD
Polymalformative syndromes	123	NRD
Neurodegeneration	123	NRD
Congenital eye defects	107	OERD
Nephropathy	90	NRD
Corneal Dystrophy	80	OERD
Metabolic	65	NRD
Myopathy	63	NRD
**TOTAL**	**5683**	

Table S8. Gene panel used in the diagnosis of cases with inherited retinal dystrophies (IRD).

**Gene**				
ABCA4	GUCY2D	RBP3	BBS9	NR2F1
ABHD12	HGSNAT	RBP4	C5orf42	OTX2
ADAM9	HK1	RCBTB1	CC2D2A	PANK2
ADAMTS18	HMCN1	RD3	CDH23	PAX2
ADGRV1	IDH3B	RDH12	CDH3	PCDH15
ADIPOR1	IFT140	RDH5	CEP164	PDZD7
AFG3L2	IFT172	RGR	CEP41	PEX1
AHR	IMPDH1	RGS9	CIB2	PEX2
AIPL1	IMPG2	RGS9BP	CISD2	PEX6
ARL3	ITM2B	RHO	CLN3	PEX7
ARL6	KCNJ13	RIMS1	CLN5	PGK1

ATF6	KCNV2	RLBP1	CLN6	PHYH
BBS1	KLHL7	ROM1	CLN8	PNPLA6
BBS2	LCA5	RP1	CNNM4	POMGNT1
BEST1	LRAT	RP1L1	COL11A1	PPT1
C2orf71	LRIT3	RP2	COL11A2	PRPS1
C8orf37	MAK	RP9	COL2A1	RPGRIP1L
CA4	MERTK	RPE65	COL9A1	SDCCAG8
CABP4	MFRP	RPGR	COL9A2	SLC41A1
CACNA1F	MFSD8	RPGRIP1	COL9A3	SLC9A6
CACNA2D4	MKKS	RS1	CTSD	SPG7
CDHR1	MKS1	SAG	DNAJC5	TCTN1
CEP290	NDP	SEMA4A	DYNC2H1	TCTN2
CERKL	NEUROD1	SLC24A1	FLVCR1	TIMM8A
CHM	NMNAT1	SNRNP200	GALE	TMEM138
CLRN1	NR2E3	SPATA7	GLIS2	TMEM216
CNGA1	NRL	TEAD1	GNPTG	TMEM237
CNGA3	NYX	TIMP3	GRN	TMEM67
CNGB1	OAT	TMEM126A	HARS	TPP1
CNGB3	OFD1	TOPORS	HMX1	TREX1
CRB1	OPA1	TRPM1	IFT80	TRIM32
CRX	OPA3	TSPAN12	INPP5E	TTC21B
CYP4V2	OPN1SW	TTC8	INVS	TUB
DHDDS	PAX6	TTPA	IQCB1	TUBGCP6
EFEMP1	PDE6A	TULP1	JAG1	UNC119
ELOVL4	PDE6B	USH2A	KIF11	USH1C
EYS	PDE6C	ZNF513	KIF7	USH1G
FAM161A	PDE6G	AHI1	LAMA1	VCAN
FSCN2	PDE6H	ALMS1	LRP5	VPS13B
FZD4	PITPNM3	ALDH3A2	LZTFL1	WDPCP
GDF6	PLA2G5	ANTXR1	MFN2	WDR19
GNAT1	PRCD	ARL13B	MKKS	WFS1
GNAT2	PROM1	ATXN7	MTTP	WHRN
GNB3	PRPF3	B9D1	MVK	XPNPEP3
GPR179	PRPF31	B9D2	MYO7A	ZNF423
GRK1	PRPF6	BBS10	NBAS	
GRM6	PRPF8	BBS12	NEK8	

GUCA1A	PRPH2	BBS4	NPHP1
GUCA1B	RAX2	BBS5	NPHP3
	RB1	BBS7	NPHP4

Table S9. Genes classified as involved in other eye related diseases (OERD). The list includes all genes linked with the HPO term “Eye Disease” – HP:0000478, but those included in the Table S8.

Gene							
AAAS	CENPJ	EPHX2	HSD3B7	MYH3	PRKG1	SPEF2	ZNF469
AARS2	CEP152	EPM2A	HSF4	MYH8	PRMT7	SPG11	ZNF644
AASS	CEP57	ERBB3	HSPD1	MYH9	PRNP	SPINT2	
ABCA1	CEP85L	ERCC1	HSPG2	MYLK	PROC	SPR	
ABCA12	CFH	ERCC2	HTRA1	MYO18B	PROK2	SPRED1	
ABCA2	CFHR1	ERCC3	HTT	MYO5A	PROKR2	SPRY4	
ABCA7	CFHR3	ERCC4	HUWE1	MYO6	PROP1	SPTBN2	
ABCB7	CFI	ERCC5	HYDIN	MYO9A	PROS1	SQSTM1	
ABCC6	CFL2	ERCC6	HYLS1	MYOC	PRRX1	SRC	
ABCC8	CHAT	ERCC8	ICOS	MYOD1	PRSS12	SRCAP	
ABCC9	CHD3	ERLIN2	IDS	MYPN	PRSS56	SRD5A3	
ABCD1	CHD7	ERMARD	IDUA	MYT1L	PRX	SREBF1	
ABCD4	CHD8	ESCO2	IER3IP1	NAA10	PSAP	SRPX2	
ABCG8	CHEK2	ESPN	IFIH1	NAGA	PSAT1	SRY	
ABHD5	CHMP1A	ESR1	IFITM5	NARS2	PSEN1	ST14	
ABL1	CHMP4B	ETFA	IFNG	NCF1	PSEN2	ST3GAL3	
ACADS	CHN1	ETFB	IFT122	NCF2	PTCH1	ST3GAL5	
ACADSB	CHRDL1	ETFDH	IFT43	NCF4	PTCH2	STAT3	
ACBD5	CHRNA1	ETHE1	IGBP1	NDE1	PTF1A	STAT4	
ACO2	CHRNA3	EVC	IGF1	NDN	PTH	STIM1	
ACOX1	CHRNA7	EVC2	IGF1R	NDRG1	PTH1R	STOX1	
ACSL4	CHRNB1	EXOSC3	IGF2	NDST1	PTPN11	STRA6	
ACTA1	CHRND	EXOSC9	IGFBP7	NDUFA1	PTPN22	STRADA	
ACTB	CHRNE	EXT2	IGLL1	NDUFA10	PTPN23	STS	
ACTG1	CHRNG	EXTL3	IGSF3	NDUFA11	PTS	STT3A	
ACTN2	CHST3	EYA1	IKZF1	NDUFA12	PUF60	STT3B	
ACVR1	CHST6	EZH2	IL10	NDUFA13	PUS1	STX11	
ACY1	CHSY1	FA2H	IL11RA	NDUFA2	PUS3	STX16	
ADAM17	CIC	FAM126A	IL12B	NDUFA4	PWRN1	STXBP1	
ADAM22	CITED2	FAM20C	IL17F	NDUFA6	PXDN	SUCLA2	
ADAMTS10	CLCC1	FAN1	IL17RA	NDUFA9	PYCR1	SUFU	
ADAMTS17	CLCN2	FANCA	IL1RAPL1	NDUFAF1	RAB18	SUMF1	

ADAMTS2	CLCN4	FANCB	IL23R	NDUFAF2	RAB23	SUOX
ADAMTSL4	CLCN7	FANCC	IL2RA	NDUFAF4	RAB27A	SUZ12
ADAR	CLCNKB	FANCD2	IL6	NDUFAF5	RAB28	SYNE1
ADARB1	CLDN16	FANCE	IL6ST	NDUFAF6	RAB39B	SYNE2
ADGRG1	CLDN19	FANCF	INPP5K	NDUFB11	RAB3GAP1	SYNGAP1
ADK	CLEC7A	FANCG	INPPL1	NDUFB3	RAB3GAP2	SYT14
ADNP	CNBP	FANCI	INS	NDUFB9	RAC1	SYT2
ADSL	CNKSR2	FANCL	INSR	NDUFS1	RAD21	TAB2
AGA	CNOT3	FANCM	IPW	NDUFS2	RAD50	TAC3
AGK	CNTN1	FARS2	IQSEC2	NDUFS4	RAD51	TACO1
AGL	COA3	FAS	IRX5	NDUFS6	RAD51C	TACR3
Aug-02	COG1	FASLG	ISCA2	NDUFS7	RAF1	TACSTD2
AGRN	COG4	FASTKD2	ITCH	NDUFV1	RAG1	TAF1
AGTPBP1	COG5	FBLN1	ITGA2B	NDUFV2	RAG2	TAF2
AGXT	COG6	FBLN5	ITGA3	NEB	RAI1	TARDBP
AHCY	COL12A1	FBN2	ITGA7	NEDD4L	RALGAPA1	TAT
AHSG	COL17A1	FBXO11	ITGB3	NEFL	RANBP2	TBC1D23
AIFM1	COL18A1	FBXO7	ITGB6	NEK1	RAPSN	TBC1D24
AIMP1	COL1A1	FBXW11	ITPA	NF1	RARS2	TBCD
AIP	COL1A2	FDFT1	ITPR1	NFIX	RASGRP1	TBCE
AIRE	COL25A1	FERMT1	JAK2	NFKB2	RAX	TBK1
AKT1	COL4A1	FGD1	JAM3	NGF	RBBP8	TBL1XR1
AKT3	COL4A2	FGF10	KANK1	NGLY1	RBM10	TBP
ALDH18A1	COL4A3	FGF14	KANSL1	NHLRC1	RBM8A	TBX1
ALDH5A1	COL4A4	FGF20	KAT5	NHP2	RBPJ	TBX15
ALDH6A1	COL4A5	FGF3	KAT6A	NHS	RECQL4	TBX2
ALDH7A1	COL4A6	FGF8	KAT6B	NIN	RELN	TBX22
ALDOA	COL5A1	FGF9	KBTBD13	NIPAL4	RFT1	TBX4
ALDOB	COL5A2	FGFR1	KCNA1	NIPBL	RIC1	TCF3
ALG1	COL6A1	FGFR2	KCNA4	NKX2-5	RIN2	TCF4
ALG11	COL6A2	FGFR3	KCNC3	NKX2-6	RIPK4	TCIRG1
ALG12	COL6A3	FGFRL1	KCND3	NKX3-2	RNASEH2A	TCOF1
ALG13	COL7A1	FH	KCNJ1	NLRP1	RNASEH2B	TCTN3
ALG14	COL8A2	FHL1	KCNJ11	NLRP3	RNASET2	TDO2
ALG2	COLEC10	FIG4	KCNJ2	NME8	RNF168	TDP1
ALG3	COLEC11	FKBP10	KCNJ6	NOD2	ROBO3	TDRD7
ALG6	COLQ	FKBP14	KCNMA1	NODAL	ROR2	TECR
ALG8	COMT	FKRP	KCNN3	NOG	RORA	TEK
ALG9	COQ2	FKTN	KCNQ3	NOP56	RPIA	TELO2
ALOX12B	COQ5	FLCN	KCNQ5	NOTCH1	RPL11	TERT
ALOXE3	COQ8A	FLI1	KCTD7	NOTCH2	RPL35A	TET2

ALPL	CORIN	FLNA	KDM5C	NOTCH3	RPL5	TFAP2A
ALS2	COX10	FLNB	KDM6A	NPAP1	RPS10	TFAP2B
ALX1	COX14	FLT4	KDM6B	NPC1	RPS19	TFG
ALX3	COX15	FMN2	KERA	NPC2	RPS24	TFRC
ALX4	COX6B1	FMR1	KIF1A	NPM1	RPS26	TGFB1
AMACR	COX7B	FOXC1	KIF1B	NRAS	RPS28	TGFB2
AMER1	CP	FOXC2	KIF21A	NRXN1	RPS6KA3	TGFB3
ANAPC1	CPT2	FOXE3	KIF5A	NSD1	RPS7	TGFBI
ANK1	CR2	FOXG1	KISS1	NSD2	RRAS2	TGIF1
ANKH	CRADD	FOXJ1	KISS1R	NSDHL	RRM2B	TGM1
ANKRD11	CRBN	FOXP1	KIT	NSMF	RSPH4A	TGM5
ANO10	CREBBP	FOXRED1	KITLG	NSUN2	RSPH9	TGM6
ANOS1	CRLF1	FRAS1	KLF11	NTF4	RSRC1	TH
AP1S1	CRTAP	FREM1	KLHL41	NTRK1	RTTN	THOC2
AP1S2	CRYAA	FREM2	KMT2C	NTRK2	RUBCN	THPO
AP3B1	CRYAB	FRG1	KMT2D	NUBPL	RUNX2	THRA
AP3B2	CRYBA1	FRMD7	KMT2E	NUP62	SACS	THRB
AP3D1	CRYBA4	FRMPD4	KNL1	OCA2	SALL1	TK2
AP4B1	CRYBB1	FTL	KRAS	OCLN	SALL4	TMCO1
AP4E1	CRYBB2	FUCA1	KRIT1	OCRL	SAMD9	TMEM231
AP4S1	CRYBB3	FUS	KRT1	ODC1	SAMHD1	TMEM70
AP5Z1	CRYGB	FUT8	KRT10	OGT	SAR1B	TMTC3
APOA1	CRYGC	FXN	KRT12	OPHN1	SARDH	TNFAIP3
APOC2	CRYGD	FYCO1	KRT14	OPN1LW	SATB2	TNFRSF11A
APOE	CRYGS	GABRA1	KRT3	OPN1MW	SBDS	TNFRSF11B
APP	CSF1R	GABRA2	KRT5	OPTN	SBF2	TNFRSF13B
APTX	CSGALNACT1	GABRA5	KRT74	ORAI1	SC5D	TNFRSF13C
ARHGAP31	CST3	GABRB3	KRT83	ORC1	SCARF2	TNFRSF1A
ARHGDIA	CST6	GABRD	KRT86	OSMR	SCN1A	TNFSF11
ARID1A	CSTA	GABRG2	L1CAM	OSTM1	SCN1B	TNFSF4
ARID1B	CTC1	GAD1	L2HGDH	P3H1	SCN2A	TOR1A
ARSA	CTDP1	GALC	LAMA2	P3H2	SCN3A	TP53RK
ARSB	CTLA4	GALK1	LAMA3	PACS1	SCN4A	TP63
ARX	CTNNB1	GALNS	LAMB1	PAFAH1B1	SCN8A	TPI1
ASAH1	CTNND1	GALNT2	LAMB2	PAH	SCN9A	TPM2
ASB10	CTNS	GALNT3	LAMB3	PARK7	SCP2	TPM3
ASNS	CTSA	GALT	LAMC2	PAX1	SCYL1	TRAF3IP1
ASPA	CTSK	GAN	LAMC3	PAX3	SDHA	TRAF3IP2
ASPM	CYB5A	GAS2L2	LAMP2	PAX4	SDHAF1	TRAK1
ASXL1	CYB5R3	GATA1	LARGE1	PAX7	SEC23A	TRAPPC2
ATCAY	CYBA	GATA2	LARS2	PBX1	SEC23B	TRAPPC9

ATIC	CYBB	GATA3	LAS1L	PCK1	SELENON	TREM2
ATM	CYP1B1	GATA4	LBR	PCLO	SEMA3A	TRIM37
ATN1	CYP24A1	GATA5	LCAT	PCNT	SEMA3E	TRIO
ATOH7	CYP27A1	GATA6	LDLRAP1	PDCD1	SEPSECS	TRIP11
ATP13A2	CYP7B1	GBA	LEMD3	PDCD10	SERAC1	TRIP12
ATP1A2	DAG1	GCDH	LETM1	PDE4D	SERPINC1	TRMT1
ATP1A3	DARS2	GCH1	LGI4	PDGFB	SERPING1	TRPV3
ATP2B2	DBH	GCK	LHX3	PDGFRB	SERPINH1	TRPV4
ATP2B3	DCC	GCNT2	LHX4	PDHA1	SERPINI1	TRRAP
ATP6AP1	DCN	GDF3	LIFR	PDHB	SETBP1	TSEN2
ATP6V0A2	DCTN1	GDF5	LIG4	PDHX	SETD2	TSEN34
ATP8A2	DCX	GDNF	LIM2	PDP1	SETX	TSFM
ATR	DDB2	GEMIN4	LINS1	PDSS1	SF3B1	TSHR
ATRX	DDC	GFM1	LIPH	PDSS2	SF3B4	TSR2
ATXN1	DDHD2	GFPT1	LMBR1	PDX1	SH2B3	TTBK2
ATXN10	DDOST	GGCX	LMNB1	PDXK	SH3BP2	TTC19
ATXN2	DDR2	GHR	LMOD3	PDYN	SH3PXD2B	TTC37
ATXN3	DDX11	GIGYF2	LMX1B	PEPD	SH3TC2	TTI2
ATXN8OS	DDX3X	GJA1	LOX	PEX10	SHANK3	TTR
AUH	DDX58	GJA3	LOXL1	PEX11B	SHH	TUBB2B
AUTS2	DEAF1	GJA5	LPL	PEX12	SHOC2	TUBB3
AVP	DGCR2	GJA8	LRBA	PEX13	SHROOM4	TUBB6
B3GALNT2	DGCR6	GJB1	LRP1	PEX14	SIGMAR1	TUBGCP4
B3GLCT	DGCR8	GJB2	LRP2	PEX16	SIK3	TUSC3
B4GALNT1	DGUOK	GJB3	LRP4	PEX19	SIL1	TWIST1
B4GALT7	DHCR24	GJB6	LRPAP1	PEX26	SIX1	TWIST2
BANF1	DHCR7	GJC2	LRPPRC	PEX3	SIX3	TWNK
BAP1	DHODH	GK	LRRC8A	PEX5	SIX6	TXN2
BCAP31	DHX37	GLB1	LRRK2	PGAP2	SKI	TYMP
BCL10	DIAPH1	GLE1	LTBP2	PGAP3	SLC12A3	TYR
BCL11A	DICER1	GLI1	LTBP4	PHF21A	SLC12A6	TYRP1
BCOR	DIS3L2	GLI2	LYST	PHF6	SLC16A12	UBA1
BCORL1	DKC1	GLI3	MADD	PHGDH	SLC16A2	UBA5
BCR	DLAT	GLIS3	MAF	PHIP	SLC17A5	UBE2A
BCS1L	DLD	GLRB	MAFA	PHOX2A	SLC19A2	UBE3A
BEAN1	DLG3	GLRX5	MAFB	PHOX2B	SLC19A3	UBIAD1
BFSP1	DLG4	GM2A	MAGEL2	PI4KA	SLC1A2	UBR1
BFSP2	DLL1	GNAI3	MALT1	PIEZO2	SLC1A3	UCHL1
BGN	DLX5	GNAQ	MAN1B1	PIGA	SLC20A2	UGP2
BIN1	DMPK	GNAS	MAN2B1	PIGL	SLC24A5	UGT1A1
BLK	DNAAF1	GNB5	MANBA	PIGN	SLC25A1	UMPS

BLM	DNAAF2	GNE	MAP2K1	PIGO	SLC25A13	UNC80
BLNK	DNAAF3	GNPAT	MAP2K2	PIGV	SLC25A15	UQCRFS1
BLOC1S5	DNAAF5	GNPTAB	MAPK1	PIK3CA	SLC25A19	UROC1
BLOC1S6	DNAH11	GNRH1	MAPKAPK3	PIK3CD	SLC25A20	UROS
BMP1	DNAH5	GNRHR	MAPT	PIK3R1	SLC25A22	USB1
BMP2	DNAH9	GORAB	MARS2	PIK3R2	SLC25A4	USP7
BMP4	DNAI1	GP1BA	MASP1	PIK3R5	SLC26A2	USP9X
BMPER	DNAI2	GP1BB	MBD5	PIKFYVE	SLC29A3	VANGL2
BNC2	DNAJC19	GPC3	MBOAT7	PINK1	SLC2A1	VAX1
BOLA3	DNAJC6	GPC4	MBTPS2	PITX1	SLC2A10	VCP
BRAF	DNAL1	GPIHBP1	MCM3AP	PITX2	SLC33A1	VIM
BRAT1	DNASE1L3	GPR143	MCM5	PITX3	SLC35A1	VLDLR
BRIP1	DNM1	GRHL2	MCOLN1	PLA2G6	SLC35A2	VMA21
BRPF1	DNM1L	GRIA3	MECP2	PLCB4	SLC35C1	VPS13A
BTK	DNM2	GRID2	MED12	PLCD1	SLC36A2	VPS35
BUB1	DNMT1	GRIK2	MED13	PLCG2	SLC37A4	VSX1
BUB1B	DNMT3A	GRIN1	MED13L	PLEC	SLC39A13	VSX2
C19orf12	DNMT3B	GRIN2A	MED23	PLEKHG2	SLC39A4	WAS
C1QTNF5	DOCK3	GRIN2B	MED25	PLEKHM1	SLC3A1	WASHC4
C1R	DOCK6	GRIP1	MEF2C	PLG	SLC40A1	WASHC5
C9orf72	DOCK7	GRM1	MEFV	PLOD1	SLC45A2	WDFY3
CA2	DOCK8	GRM7	MEGF8	PLOD3	SLC4A11	WDR11
CA8	DOK7	GSC	MEIS2	PLP1	SLC4A4	WDR35
CACNA1A	DPAGT1	GSN	METTL5	PMM2	SLC52A2	WDR36
CACNA1D	DPM1	GSR	MFF	PMP22	SLC52A3	WDR4
CACNA1E	DPP6	GSS	MGP	PMS1	SLC5A7	WDR45
CACNA1G	DPYD	GTF2H5	MID1	PNKD	SLC6A19	WDR45B
CACNA1H	DSE	GUSB	MIF	PNPLA1	SLC6A20	WDR81
CACNA2D2	DSG4	HACE1	MIP	PNPO	SLC6A3	WIPF1
CACNB4	DST	HADH	MIR184	PNPT1	SLC6A8	WNK1
CACNG2	DTNBP1	HADHA	MITF	POC1A	SLC6A9	WNT10A
CALR	DVL1	HADHB	MKRN3	POGZ	SLCO2A1	WNT10B
CAMK2G	DYNC1H1	HBA2	MLH3	POLD1	SLITRK6	WNT3
CAMTA1	DYNC2LI1	HBB	MLPH	POLG	SLX4	WNT5A
CANT1	DYRK1A	HCCS	MLXIPL	POLG2	SMARCA4	WRAP53
CASK	EARS2	HCN1	MMADHC	POLH	SMARCAL1	WRN
CASP10	EBP	HCRT	MME	POLR1C	SMARCB1	WWOX
CASR	EDN1	HDAC4	MMP1	POLR1D	SMARCE1	XPA
CAV1	EDN3	HDAC6	MMP14	POLR3A	SMC1A	XPC
CBL	EDNRA	HDAC8	MMP2	POLR3B	SMC3	XRCC1
CBS	EDNRB	HERC2	MN1	POMGNT2	SMCHD1	XRCC2

CC2D1A	EFEMP2	HESX1	MOCS1	POMK	SMO	XRCC4
CCBE1	EFHC1	HEXA	MOCS2	POMT1	SMOC1	XYLT1
CCDC22	EFNB1	HEXB	MOGS	POMT2	SMPD1	XYLT2
CCDC39	EGFR	HGD	MPC1	POR	SMS	YAP1
CCDC40	EGR2	HIBCH	MPDZ	PORCN	SNAI2	YARS2
CCDC88C	EHMT1	HLCS	MPL	POU1F1	SNAP25	YIF1B
CCM2	EIF2AK3	HMGA2	MPV17	POU3F4	SNAP29	YY1
CCND1	EIF2B1	HNF4A	MPZ	POU6F2	SNCA	ZBTB16
CD19	EIF2B2	HNMT	MRE11	PPIB	SNIP1	ZBTB18
CD247	EIF2B3	HNRNPU	MSMO1	PPM1D	SNORD115-1	ZBTB24
CD27	EIF2B4	HOXA1	MSX2	PPP1R17	SNORD116-1	ZC3H14
CD79A	EIF2B5	HOXA13	MTAP	PPP2R1A	SNRPN	ZDHHC9
CD79B	EIF2S3	HOXB1	MTFMT	PPP2R2B	SNX10	ZEB1
CD96	EIF4G1	HPGD	MTHFR	PPP3CA	SOBP	ZEB2
CDC42	ELN	HPS1	MTHFS	PQBP1	SOS1	ZFHX4
CDH1	ELP4	HPS3	MTM1	PRDM16	SOST	ZFPM2
CDH15	EMD	HPS4	MTMR14	PRDM5	SOX2	ZFYVE26
CDK4	ENPP1	HPS5	MTO1	PRDX1	SOX3	ZIC1
CDK5RAP2	ENTPD1	HPS6	MTPAP	PREPL	SOX5	ZIC2
CDKL5	EP300	HRAS	MTR	PRF1	SOX6	ZIC3
CDKN1C	EPAS1	HS2ST1	MTRR	PRICKLE3	SOX9	ZMPSTE24
CDKN2A	EPB41L1	HS6ST1	MUSK	PRKAR1A	SPAST	ZMYND11
CDON	EPCAM	HSD11B2	MYF5	PRKCG	SPATA5	ZNF335
CEL	EPHA2	HSD17B4	MYH2	PRKDC	SPECC1L	ZNF365

Table S10. Genes in the database of allelic frequencies not involved in an eye related disease (NRD).

Gene						
A2M	CCRL2	EPOR	IDH2	MSH3	PRMT3	SRGAP2
A4GALT	CCT5	EPX	IDO1	MSH4	PRMT9	SRGAP3
A4GNT	CD109	ERAP1	IFI30	MSMB	PRND	SRI
AADAC	CD14	ERAP2	IFI44L	MSR1	PRODH	SRP72
AADACL2	CD151	ERBB2	IFITM3	MSRA	PROK1	SRPX
AAGAB	CD177	ERBB4	IFNA10	MSRB3	PROKR1	SRR
AARS	CD1A	ERI2	IFNA17	MST1R	PROX1	SSH1
ABAT	CD1E	ERMAP	IFNA2	MSTN	PROZ	SSPN
ABCA10	CD200	ERRFI1	IFNAR1	MSX1	PRPH	SST
ABCA13	CD207	ESAM	IFNAR2	MT1A	PRSS1	SSTR5
ABCA3	CD209	ESR2	IFNGR1	MT2A	PRSS8	SSX7

ABCB1	CD22	ESRRB	IFNGR2	MTA1	PRTG	ST3GAL1
ABCB11	CD226	ESRRG	IFNL3	MTA2	PSCA	ST3GAL2
ABCB4	CD244	ETNPPL	IFRD1	MTCH2	PSMA6	ST3GAL4
ABCC1	CD2AP	ETS1	IFT88	MTHFD1	PSMC2	ST3GAL6
ABCC11	CD320	ETV4	IGF2BP2	MTHFD1L	PSMC3IP	ST5
ABCC2	CD36	ETV6	IGF2R	MTMR2	PSMD7	ST6GAL1
ABCC3	CD38	EVI5	IGFALS	MTMR9	PSPH	ST6GAL2
ABCC4	CD3D	EWSR1	IGFBP1	MTNR1A	PSTPIP1	ST6GALNAC1
ABCD3	CD3E	EXO1	IGFBP3	MTNR1B	PSTPIP2	ST6GALNAC2
ABCG1	CD3G	EXO5	IGFBP5	MTSS1	PTAFR	ST6GALNAC3
ABCG2	CD4	EXOC4	IGHMBP2	MTUS1	PTCHD1	ST6GALNAC4
ABI3BP	CD40	EXPH5	IGSF1	MUC1	PTCHD3	ST6GALNAC5
ABL2	CD40LG	EXT1	IHH	MUC13	PTCSC3	ST6GALNAC6
ABO	CD44	EXTL1	IKBIP	MUC15	PTGDR	ST7
ACACA	CD46	EXTL2	IKBKAP	MUC2	PTGDR2	ST8SIA1
ACACB	CD5	EYA4	IKBKB	MUC3A	PTGDS	ST8SIA2
ACAD10	CD55	F10	IKZF3	MUC4	PTGER2	ST8SIA3
ACAD11	CD58	F11	IL10RA	MUC5B	PTGER4	ST8SIA4
ACAD8	CD59	F12	IL10RB	MUC6	PTGES2	ST8SIA5
ACAD9	CD72	F13A1	IL11	MUC7	PTGIR	ST8SIA6
ACADL	CD74	F13B	IL12A	MURC	PTGIS	STAR
ACADM	CD80	F2	IL12RB1	MUS81	PTGS1	STARD9
ACADVL	CD81	F2R	IL12RB2	MUT	PTGS2	STAT1
ACAN	CD86	F2RL1	IL13	MX1	PTHLH	STAT5B
ACAT1	CD8A	F3	IL16	MXI1	PTK7	STAT6
ACAT2	CDA	F5	IL17A	MYB	PTPN1	STEAP3
ACBD6	CDAN1	F7	IL17RB	MYBL2	PTPN12	STEAP4
ACCS	CDC42BPB	F8	IL17REL	MYBPC1	PTPN13	STH
ACE	CDC6	F9	IL18	MYC	PTPN14	STIL
ACHE	CDC73	FAAH	IL18R1	MYCL	PTPN2	STK10
ACKR1	CDCA7L	FAAH2	IL18RAP	MYCN	PTPN21	STK11IP
ACLY	CDH12	FABP1	IL19	MYEF2	PTPN6	STK3
ACP1	CDH13	FABP2	IL1A	MYF6	PTPRB	STK32A
ACP5	CDH5	FABP3	IL1B	MYH13	PTPRC	STK33
ACSF3	CDH8	FABP4	IL1R1	MYH14	PTPRD	STK35
ACSL5	CDK11A	FABP6	IL1RL1	MYH15	PTPRF	STK36
ACSL6	CDK16	FABP7	IL1RN	MYH6	PTPRJ	STK39
ACSM2B	CDK5R1	FADD	IL2	MYL1	PTPRK	STK4

ACSM3	CDK5RAP3	FADS2	IL20RA	MYLIP	PTPRN2	STMN1
ACTN3	CDK6	FAH	IL20RB	MYLK2	PTPRO	STRC
ACTN4	CDK7	FAM104A	IL21	MYO15A	PTPRQ	STX1A
ACVR1B	CDKAL1	FAM120A	IL21R	MYO1A	PTPRT	STXBP2
ACVR1C	CDKL3	FAM134B	IL2RG	MYO1C	PTRF	STXBP5
ACVR2A	CDKN1A	FAM161B	IL3	MYO1E	PUS10	SUCLG1
ACVR2B	CDKN1B	FAM205A	IL31RA	MYO1F	PVR	SUCO
ADA	CDKN2B	FAM20A	IL36RN	MYO3A	PVT1	SUGCT
ADAM10	CDKN2B-AS1	FAM47B	IL4	MYO5B	PYCRL	SULF1
ADAM12	CDKN2C	FAM58A	IL4R	MYO5C	PYGB	SULT1A1
ADAM19	CDT1	FAM83H	IL5	MYO7B	PYGL	SULT1C2
ADAM23	CDX2	FAM8A1	IL6R	MYO9B	PYGM	SULT1E1
ADAM33	CEACAM16	FAM91A1	IL7	MYOCD	PYY	SULT2A1
ADAM7	CEBPA	FASN	IL7R	MYOM1	PZP	SULT2B1
ADAMTS1	CEBPE	FBLIM1	IL9	MYOT	QDPR	SULT4A1
ADAMTS13	CECR2	FBN3	ILDR1	MYOZ2	QKI	SUMO1
ADAMTS16	CELSR1	FBP1	ILK	MYT1	RAB11FIP5	SUMO4
ADAMTSL2	CELSR2	FBXO10	IMMP2L	NAGLU	RAB25	SUN2
ADAMTSL3	CEMIP	FBXO18	IMMT	NAGPA	RAB27B	SUPT16H
ADCY10	CENPO	FBXW4	IMPA2	NAIP	RAB29	SV2B
ADCY3	CENPP	FBXW7	IMPAD1	NAMPT	RAB2A	SYCE2
ADCY5	CEP135	FCAR	IMPDH2	NAT1	RAB40AL	SYCP3
ADCY6	CEP63	FCER1A	INF2	NAT2	RAB7A	SYK
ADCY9	CEP68	FCER2	ING1	NAT8L	RABGGTA	SYN1
ADCYAP1	CER1	FCGR1A	INMT	NAV2	RABL6	SYN2
ADD1	CERS6	FCGR2A	INPP4A	NBEA	RAC2	SYN3
ADD2	CES1	FCGR2B	INPP5B	NBEAL2	RAD21L1	SYNGR1
ADH1B	CES2	FCGR3A	INPP5D	NBN	RAD23B	SYNM
ADH1C	CETP	FCGR3B	INSIG1	NBPF1	RAD51B	SYNPO
ADH4	CFAP53	FCGRT	INSIG2	NCALD	RAD51D	SYP
ADH5	CFAP57	FCN2	INSL3	NCAM1	RAD52	SYT11
ADH7	CFC1	FCN3	INSL6	NCAN	RAD54B	SYTL3
ADIPOQ	CFD	FCRL3	IQGAP1	NCAPD2	RAD54L	SYTL5
ADM	CFHR2	FECH	IQGAP2	NCKAP1	RAD9A	T
ADORA1	CFHR4	FEM1A	IQGAP3	NCOA1	RAET1L	TAAR1
ADORA2A	CFHR5	FEM1B	IRAK1	NCOA3	RALGDS	TAAR6
ADORA3	CFLAR	FEN1	IRAK3	NCOA4	RANGRF	TAAR9
ADRA1A	CFP	FERMT3	IRAK4	NCS1	RAP1GDS1	TAF15

ADRA2A	CFTR	FEV	IRF1	NCSTN	RARA	TAF1C
ADRA2B	CGA	FEZF2	IRF2	NDOR1	RASA1	TAF1L
ADRA2C	CGB3	FFAR1	IRF4	NDST2	RASGRP2	TAF7L
ADRB1	CHD1L	FFAR4	IRF5	NDST3	RASSF1	TAL1
ADRB2	CHD2	FGA	IRF6	NDST4	RASSF5	TAL2
ADRB3	CHD6	FGB	IRF8	NDUFA7	RB1CC1	TALDO1
ADTRP	CHDH	FGD3	IRGM	NDUFA8	RBFOX1	TAS1R1
AFF2	CHFR	FGD4	IRS1	NDUFAF7	RBL1	TAS1R2
AFF3	CHGA	FGF1	IRS2	NDUFB1	RBL2	TAS2R16
AFP	CHGB	FGF2	IRS4	NDUFB6	RBM15	TAS2R3
AGBL4	CHI3L1	FGF23	IRX4	NDUFS5	RBM20	TAS2R38
AGGF1	CHI3L2	FGFBP1	ISCU	NDUFV3	RBM28	TAS2R9
AGMO	CHIA	FGFR4	ISL1	NEBL	RBMXL2	TAZ
AGO1	CHIC2	FGG	ISPD	NECTIN1	RC3H1	TBC1D1
AGPAT2	CHIT1	FHIT	ITGA1	NECTIN4	RCAN1	TBC1D4
AGPS	CHL1	FHL2	ITGA11	NEDD4	RDH8	TBC1D9
AGRP	CHMP2B	FIGLA	ITGA2	NEDD9	RDX	TBL1X
AGT	CHPF2	FIP1L1	ITGA4	NEFH	REEP1	TBL1Y
AGTR1	CHRD	FKBP1A	ITGA6	NEFM	REL	TBX10
AGTR2	CHRFAM7A	FKBP4	ITGA9	NEGR1	REN	TBX19
AGXT2	CHRM1	FKBP5	ITGAE	NEIL1	REPS2	TBX20
AHRR	CHRM2	FKBP6	ITGAM	NEIL2	RETN	TBX21
AHSP	CHRM3	FKBP8	ITGB1	NELFA	REV3L	TBX3
AICDA	CHRNA2	FLG	ITGB2	NELL1	RFC2	TBX5
AK1	CHRNA4	FLT1	ITGB4	NEU2	RFWD2	TBXA2R
AK2	CHRNA5	FLT3	ITIH1	NEUROG3	RFX2	TBXAS1
AK7	CHRNA9	FLVCR2	ITIH3	NEXN	RFX5	TCF21
AK8	CHRNB2	FMN1	ITIH4	NFATC2	RFX6	TCF7
AKAP10	CHRNB4	FMO1	ITIH6	NFATC3	RFX8	TCF7L1
AKAP13	CHST7	FMO2	ITK	NFATC4	RFXANK	TCF7L2
AKAP9	CHST8	FMO3	ITPKC	NFE2L1	RFXAP	TCN1
AKR1B1	CHSY3	FMO4	ITPR3	NFE2L2	RGMA	TCN2
AKR1C2	CHUK	FMO5	ITSN2	NFIA	RGS2	TCP1
AKR1C3	CIAPIN1	FMO6P	IVD	NFKB1	RGS6	TCTE1
AKR1C4	CIDEA	FMOD	IYD	NFKBIA	RGS7	TCTE3
AKR1D1	CIDEC	FN1	JAG2	NFKBIZ	RHAG	TDGF1
AKR7A2	CIITA	FN3K	JAK3	NFU1	RHBDF2	TEC
AKR7A3	CILP	FOLH1	JMJD1C	NGFR	RHCE	TECPR2

AKT2	CKM	FOLR1	JPH2	NHEJ1	RHD	TECTA
ALAD	CLCA1	FOXA2	JPH3	NICN1	RHOB	TEKT2
ALAS2	CLCA2	FOXA3	JRK	NID1	RHOH	TENM4
ALB	CLCF1	FOXD3	JUN	NINJ1	RHPN2	TEP1
ALCAM	CLCN1	FOXE1	JUNB	NIP7	RIC3	TET1
ALDH16A1	CLCN3	FOXF1	JUP	NIPA1	RIMS3	TEX14
ALDH1A1	CLCN5	FOXF2	KALRN	NIPSNAP1	RIOK2	TF
ALDH1A2	CLCN6	FOXI1	KARS	NIPSNAP3A	RMI1	TFAM
ALDH2	CLCNKA	FOXK1	KAT2A	NKAIN2	RMND1	TFB1M
ALDH4A1	CLDN1	FOXM1	KATNAL2	NKX2-1	RNASE3	TFCP2
ALG10B	CLDN14	FOXN1	KCNA3	NKX2-3	RNASEL	TFE3
ALG5	CLEC11A	FOXO1	KCNA5	NKX3-1	RNF114	TFF1
ALK	CLEC2D	FOXP2	KCNA6	NLGN1	RNF135	TFPI
ALOX12	CLEC3B	FOXP3	KCNAB1	NLGN2	RNF139	TFR2
ALOX15	CLEC4M	FPGS	KCNAB2	NLGN3	RNF170	TG
ALOX5	CLIC2	FPR1	KCND2	NLGN4X	RNF212	TGFBR3
ALOX5AP	CLIP2	FPR2	KCNE1	NLGN4Y	RNF213	TGFBRAP1
ALS2CL	CLK2	FRA10AC1	KCNE2	NLRP12	RNF6	TGM2
AMBN	CLMP	FREM3	KCNE3	NLRP14	RNLS	THADA
AMELX	CLNK	FRK	KCNE4	NLRP2	ROBO1	THAP1
AMELY	CLOCK	FRMD6	KCNE5	NLRP7	ROBO2	THBD
AMH	CLPS	FRY	KCNH3	NLRX1	ROCK1	THBS1
AMHR2	CLPTM1	FRZB	KCNIP1	NMB	ROCK2	THBS2
AMN	CLPTM1L	FSCB	KCNIP4	NME1	ROPN1L	THBS4
AMPD1	CLSTN2	FSHB	KCNJ10	NME5	ROS1	THSD7A
AMPD3	CLTCL1	FSHR	KCNJ12	NME7	RPA1	TICAM1
AMT	CLU	FST	KCNJ15	NMT2	RPH3AL	TIMM44
ANG	CLUL1	FTCD	KCNJ3	NMU	RPL21	TIMP1
ANGPT1	CLYBL	FTHL17	KCNJ5	NNT	RPL24	TIMP2
ANGPT2	CMA1	FTO	KCNJ8	NOBOX	RPL38	TINAG
ANGPTL4	CMPK1	FTSJ1	KCNJ9	NOD1	RPN2	TIRAP
ANGPTL5	CNDP1	FURIN	KCNK18	NOP16	RPS15	TJP2
ANK2	CNKSR1	FUT1	KCNK3	NOS1	RPS3	TLDC2
ANK3	CNNM2	FUT2	KCNK6	NOS1AP	RPS6KB1	TLK1
ANKK1	CNOT4	FUT3	KCNK9	NOS2	RPS6KL1	TLL1
ANKRD1	CNPY3	FUT6	KCNMB3	NOS3	RPTOR	TLR1
ANKRD26	CNR1	FUZ	KCNMB4	NPAS2	RRH	TLR10
ANKS1A	CNR2	FXYD6	KCNN2	NPAS3	RRM1	TLR2

ANKS1B	CNTF	FZD1	KCNQ2	NPAT	RRP1B	TLR3
ANKS6	CNTN4	FZD3	KCNQ4	NPC1L1	RSC1A1	TLR4
ANO3	CNTNAP2	FZD6	KCNS1	NPFFR2	RSPO1	TLR5
ANO5	CNTNAP4	FZD9	KCNS3	NPHS1	RSPO4	TLR6
ANO6	CNTNAP5	G6PC	KCNT1	NPHS2	RTN2	TLR7
ANO7	COA5	G6PC2	KCNV1	NPL	RTN4R	TLR8
ANTXR2	COCH	G6PC3	KCTD13	NPPB	RUNX1	TLR9
ANXA11	COG2	G6PD	KDM3A	NPPC	RUNX3	TLX1
ANXA5	COG3	GAB2	KDM4C	NPR1	RUVBL1	TLX2
AOAH	COG7	GABRA6	KDM5A	NPR2	RXFP2	TLX3
AOC1	COL10A1	GABRG1	KDR	NPR3	RXRA	TM4SF19
APAF1	COL6A4P2	GABRG3	KEL	NPSR1	RXRG	TMC1
APBA2	COL6A5	GABRR2	KHDC3L	NPTN	RYK	TMC6
APBB1	COMMD1	GAD2	KHK	NPY	RYR3	TMC8
APBB2	COMP	GADD45A	KIAA0100	NPY1R	S100B	TMEM114
APBB3	COQ4	GADD45B	KIAA0232	NPY2R	S1PR1	TMEM135
APCDD1	COQ9	GAK	KIAA0319	NQO1	SAA1	TMEM165
APH1A	CORO1A	GAL3ST1	KIAA0513	NQO2	SAA2	TMEM173
APH1B	COX4I1	GAL3ST2	KIAA1257	NR0B1	SAGE1	TMEM185A
APLNR	COX4I2	GAL3ST3	KIAA1462	NR0B2	SARS2	TMEM187
APOA4	COX7A2	GAL3ST4	KIAA2022	NR1H2	SART1	TMEM2
APOA5	CPA4	GALNT11	KIF17	NR1H3	SART3	TMEM249
APOBEC1	CPA6	GALNT12	KIF18A	NR1H4	SAT1	TMEM39A
APOBEC3B	CPB2	GALNT13	KIF1BP	NR1I2	SATL1	TMEM52B
APOBEC3G	CPE	GALNT14	KIF22	NR1I3	SBNO1	TMEM8A
APOBEC3H	CPLX2	GALNT18	KIF27	NR2E1	SCAP	TMEM9
APOC1	CPN1	GALNT5	KIF5B	NR2F2	SCARB1	TMEM99
APOC3	CPOX	GALNT6	KIF6	NR3C1	SCARB2	TMIE
APOD	CPS1	GALNT7	KIFAP3	NR3C2	SCG2	TMLHE
APOH	CPT1A	GALNT8	KIR2DL1	NR4A1	SCG3	TMPO
APOL1	CPT1B	GALNT9	KIR2DL3	NR4A2	SCGB1A1	TMPRSS11A
APOL3	CPZ	GALNTL5	KIR2DL4	NR4A3	SCGB1D2	TMPRSS15
APRT	CR1	GALNTL6	KIR3DL1	NR5A1	SCGB3A2	TMPRSS3
AQP1	CREB1	GALP	KIR3DL2	NRCAM	SCLT1	TMPRSS4
AQP2	CREB3L3	GAMT	KIRREL3	NRG1	SCN10A	TMPRSS5
AQP3	CRELD1	GAP43	KL	NRG3	SCN11A	TMPRSS6
AQP4	CRH	GARS	KLB	NRIP1	SCN2B	TNC
AQP5	CRHR1	GAS1	KLC1	NRP2	SCN3B	TNFAIP2

AQP7	CRISP2	GAS6	KLF10	NRTN	SCN4B	TNFRSF10A
AR	CRK	GATAD1	KLF5	NRXN2	SCN7A	TNFRSF10B
AREL1	CRKL	GATM	KLF6	NRXN3	SCNN1A	TNFRSF1B
ARFGEF2	CRP	GBA3	KLF7	NSUN7	SCNN1B	TNFRSF25
ARG1	CRYM	GBE1	KLF8	NT5C1B	SCNN1G	TNFRSF4
ARHGAP24	CSDE1	GBGT1	KLHDC8B	NT5C3A	SCO1	TNFRSF9
ARHGAP26	CSF1	GC	KLHL10	NT5E	SCRIB	TNFSF10
ARHGAP45	CSF2	GCGR	KLHL3	NTF3	SCUBE2	TNFSF13B
ARHGAP6	CSF2RB	GCKR	KLHL9	NTNG1	SDC3	TNFSF14
ARHGAP9	CSF3R	GCLC	KLK1	NTRK3	SEC63	TNFSF15
ARHGEF10	CSH1	GCLM	KLK12	NUAK1	SECISBP2	TNFSF8
ARHGEF11	CSMD1	GCM2	KLK15	NUDC	SEL1L	TNKS
ARHGEF12	CSMD3	GCNT1	KLK3	NUDT1	SELE	TNNI2
ARHGEF6	CSNK1A1L	GCSH	KLK4	NUDT6	SELL	TNNT1
ARHGEF7	CSNK1D	GDAP1	KLK7	NUMA1	SELP	TNNT3
ARHGEF9	CSNK2A2	GDF15	KLKB1	NUMBL	SELPLG	TNP1
ARID4A	CSNK2A3	GDF9	KMT5A	NUP155	SEM1	TNR
ARID4B	CSRP3	GDI1	KMT5B	NUP214	SEMA4C	TNS2
ARL11	CSTB	GEMIN2	KNG1	NXF3	SEMA4G	TNS3
ARL14EP	CTF1	GFAP	KPNA1	NXF5	SEMA6D	TNXA
ARL6IP5	CTGF	GFI1	KRT13	NXNL1	SEMA7A	TOMM40
ARMS2	CTH	GFI1B	KRT16	OAS1	SEMG1	TOMM40L
ARPC3	CTHRC1	GFPT2	KRT17	OAS2	SEPT12	TOP1
ARSE	CTNNA2	GFRA1	KRT18	OAZ1	SEPT9	TOP1MT
ARSF	CTNNA3	GFRA2	KRT2	OBSCN	SERPINA1	TOP2A
ART4	CTNND2	GGH	KRT31	OBSL1	SERPINA10	TOPBP1
ARVCF	CTRC	GGT5	KRT37	OGG1	SERPINA3	TOX3
AS3MT	CTSB	GH1	KRT38	OLFM2	SERPINA6	TP53AIP1
ASAH2	CTSC	GH2	KRT4	OLIG2	SERPINA7	TP53BP1
ASCC1	CTSG	GHRH	KRT6A	OLR1	SERPINB11	TP53I3
ASCC3	CTSZ	GHRHR	KRT6B	OPCML	SERPINB3	TP73
ASIP	CTTNBP2	GHRL	KRT6C	OPLAH	SERPINB5	TPCN2
ASL	CUBN	GHSR	KRT75	OPN4	SERPINB6	TPH1
ASPRV1	CUL2	GIF	KRT8	OPRD1	SERPIND1	TPH2
ASS1	CUL3	GIMAP8	KRT85	OPRK1	SERPINE1	TPK1
ASTN2	CUL4B	GIP	KRT9	OPRL1	SERPINF1	TPMT
ATF1	CUL5	GIPC3	KRTAP1-1	OPRM1	SERPINF2	TPO
ATF3	CUL7	GIPR	KRTCAP3	OPTC	SERPINI2	TPP2

ATG16L1	CX3CR1	GIT1	KYNU	OR10X1	SERTAD1	TPRN
ATG7	CXCL12	GJA4	L3MBTL1	OR13G1	SESN2	TPTE
ATL1	CXCL16	GJC3	LAMA4	OR1B1	SETDB2	TRADD
ATP10A	CXCL5	GJD2	LAMA5	OR51G1	SEZ6	TRAF3
ATP10D	CXCR1	GLCCI1	LAMC1	OR52H1	SEZ6L2	TRAF6
ATP13A4	CXCR3	GLDC	LAMTOR2	OR52N4	SFTPA1	TRAK2
ATP1B1	CXCR4	GLMN	LARGE2	OR5AC2	SFTPA2	TRAPPC10
ATP2A1	CYB5R4	GLO1	LBP	OR5H6	SFTPB	TREH
ATP2A2	CYBRD1	GLP1R	LCE3B	OR8K3	SFTPC	TRERF1
ATP2A3	CYCS	GLRA1	LCK	ORC4	SFTPD	TRHR
ATP2B4	CYFIP1	GLS	LCN10	ORC6	SGCA	TRIB1
ATP2C1	CYLD	GLTSCR1	LCT	OTOA	SGCB	TRIB2
ATP5E	CYP11A1	GLUD1	LDB1	OTOF	SGCD	TRIB3
ATP5SL	CYP11B1	GLUD2	LDB3	OTOG	SGCE	TRIL
ATP6AP2	CYP11B2	GLUL	LDHA	OTOGL	SGCG	TRIM17
ATP6V0A1	CYP17A1	GLYCTK	LDHB	OTOR	SGK1	TRIM21
ATP6V0A4	CYP19A1	GMIP	LDLRAD2	OVCH2	SGSH	TRIM22
ATP6V1B1	CYP1A1	GMPS	LDLRAD4	OVGP1	SH2B1	TRIM24
ATP7A	CYP1A2	GNA14	LECT2	OXCT1	SH2D1A	TRIM33
ATP8B1	CYP21A2	GNAI2	LEF1	OXTR	SH3GL1	TRIM5
ATPAF2	CYP26A1	GNAS-AS1	LEFTY2	P2RX1	SHANK2	TRIOBP
ATRIP	CYP26B1	GNB1L	LEP	P2RX4	SHBG	TRMU
ATRNL1	CYP26C1	GNS	LEPR	P2RX5	SHMT1	TROAP
ATXN3L	CYP27B1	GOLGA3	LFNG	P2RX7	SHOX2	TROVE2
AURKA	CYP2A13	GOLGA5	LGALS13	P2RY12	SHROOM3	TRPA1
AURKC	CYP2A6	GON4L	LGALS2	P2RY4	SI	TRPC3
AVPR1A	CYP2B6	GOPC	LGALS3	PABPC4L	SIAE	TRPC4
AVPR1B	CYP2C18	GOSR2	LGI1	PACRG	SIGLEC12	TRPC5
AVPR2	CYP2C19	GOT1	LGR5	PADI4	SIGLEC14	TRPC6
AXIN1	CYP2C8	GP2	LHB	PAFAH1B3	SIGLEC16	TRPM2
AXIN2	CYP2C9	GP6	LHCGR	PAK3	SIK2	TRPM3
AXL	CYP2D6	GP9	LHFPL5	PALLD	SIM1	TRPM4
AZIN2	CYP2E1	GPAM	LHX1	PAPD7	SIM2	TRPM6
B2M	CYP2F1	GPATCH8	LHX8	PAPSS2	SIPA1	TRPM7
B3GALNT1	CYP2G1P	GPBAR1	LIAS	PARD3B	SIPA1L1	TRPS1
B3GALT1	CYP2J2	GPC6	LIF	PARK2	SIRT1	TRPV1
B3GALT2	CYP2R1	GPD1	LIG1	PARL	SIRT3	TRPV5
B3GALT5	CYP2U1	GPD1L	LIG3	PARP1	SIRT5	TSG101

B3GAT1	CYP2W1	GPD2	LIMK1	PARP2	SIX2	TSHB
B3GAT2	CYP3A4	GPHN	LIN28A	PASK	SLA	TSHZ1
B3GNT2	CYP3A43	GPI	LIN28B	PAWR	SLBP	TSLP
B3GNT3	CYP3A5	GPNMB	LIPA	PAX5	SLC10A1	TSPAN17
B3GNT6	CYP3A7	GPR1	LIPC	PAX8	SLC10A2	TSPAN7
B3GNT7	CYP46A1	GPR12	LIPE	PAX9	SLC11A1	TSPEAR
B4GALNT2	CYP4A11	GPR132	LIPG	PC	SLC11A2	TSPO
B4GALNT3	CYP4A22	GPR139	LIPI	PCBD1	SLC12A1	TSSC4
B4GALNT4	CYP4B1	GPR33	LIPN	PCCA	SLC13A2	TSSK4
B4GALT1	CYP4F12	GPR55	LITAF	PCCB	SLC14A1	TTC14
B4GALT2	CYP4F2	GPR68	LLGL1	PCDH11X	SLC14A2	TTLL1
B4GALT3	CYP4F22	GPS1	LMAN1	PCDH18	SLC15A1	TTLL11
B4GALT4	CYP4F3	GPSM2	LMBRD1	PCDH19	SLC16A1	TUBA1A
B4GALT5	CYP7A1	GPX1	LMF1	PCDH9	SLC16A3	TUBA8
B4GALT6	CYS1	GPX4	LMNB2	PCDHA1	SLC17A1	TUBB1
BAALC	CYSLTR1	GRB10	LMO2	PCDHA9	SLC17A3	TUBGCP5
BAAT	CYSLTR2	GREM1	LMO4	PCDHAC1	SLC17A8	TULP3
BACE1	D2HGDH	GRHL1	LMTK3	PCDHB4	SLC18A1	TXNIP
BAG3	DAD1	GRHPR	LNX1	PCK2	SLC19A1	TXNRD2
BANK1	DAO	GRID1	LNX2	PCM1	SLC1A1	TYK2
BARD1	DAOA	GRIK1	LOC10099684 2	PCMT1	SLC1A5	TYMS
BARX2	DAPK1	GRIK3	LOR	PCOLCE	SLC22A1	TYMSOS
BAX	DAZL	GRIK4	LOXHD1	PCSK1	SLC22A11	TYRO3
BAZ1B	DBI	GRIN3A	LOXL2	PCSK2	SLC22A12	UACA
BCAM	DBT	GRK3	LOXL3	PCSK5	SLC22A14	UBA3
BCAT1	DCAF13	GRK4	LPA	PDCD5	SLC22A18	UBAC2
BCAT2	DCAF17	GRK5	LPAR1	PDE10A	SLC22A18AS	UBE2B
BCHE	DCDC2	GRM3	LPIN1	PDE11A	SLC22A2	UBE2I
BCKDHA	DCK	GRM5	LPIN2	PDE12	SLC22A3	UBE2NL
BCKDHB	DCLK1	GRM8	LPIN3	PDE4B	SLC22A4	UBE3C
BCKDK	DCLRE1C	GRPR	LPP	PDE7B	SLC22A5	UBN2
BCL2	DCP1B	GRXCR1	LRCH1	PDE8B	SLC22A6	UBQLN2
BCL2A1	DCTD	GSDMA	LRFN5	PDGFC	SLC22A9	UBR3
BCL2L1	DCXR	GSDMB	LRP6	PDGFRA	SLC23A1	UBR7
BCL2L11	DDAH1	GSE1	LRP8	PDGFRL	SLC24A2	UCP1
BCL2L2	DDHD1	GSK3B	LRRC6	PDK1	SLC25A12	UCP2
BCL6	DDX20	GSPT1	LRRFIP2	PDLIM3	SLC25A3	UCP3
BCL9	DDX25	GSPT2	LRSAM1	PDLIM4	SLC25A38	UFD1L

BCO1	DDX3Y	GSTA1	LRTOMT	PDLIM5	SLC25A39	UGCG
BDKRB2	DDX5	GSTA2	LTBP1	PDPK1	SLC26A1	UGGT1
BDNF	DDX53	GSTA3	LTBP3	PEAR1	SLC26A3	UGGT2
BEX4	DEC1	GSTK1	LTBR	PECAM1	SLC26A4	UGT1A10
BHLHA9	DECR1	GSTM1	LTF	PECR	SLC26A5	UGT1A6
BHLHE41	DEF6	GSTM3	LTK	PEMT	SLC26A6	UGT1A8
BHMT	DEFB1	GSTM4	LTN1	PENK	SLC26A9	UGT2A1
BICC1	DEFB126	GSTO1	LUM	PER1	SLC27A4	UGT2A2
BICD1	DEFB4A	GSTO2	LY96	PER2	SLC27A5	UGT2B15
BIRC5	DENND4A	GSTP1	LYN	PER3	SLC28A1	UGT2B17
BLMH	DES	GSTT1	LYZ	PFAS	SLC28A2	UGT2B28
BLVRA	DFNA5	GSTT2B	LZTS1	PFKM	SLC28A3	UGT2B4
BLZF1	DFNB59	GSTZ1	MACROD2	PGAM1	SLC29A1	UGT2B7
BMP10	DGAT1	GTF2E1	MAD1L1	PGAM2	SLC29A2	UGT8
BMP15	DGCR14	GTF2H1	MAD2L1	PGAM5	SLC29A4	UHRF1BP1
BMP2K	DGCR5	GTF2I	MAGEE2	PGBD1	SLC2A2	UIMC1
BMP5	DGKD	GTF2IRD1	MAGI2	PGC	SLC2A4	ULK4
BMP7	DGKE	GTF2IRD2	MAGT1	PGD	SLC2A9	UMOD
BMPR1B	DHFR	GUCY2C	MAML2	PGM1	SLC30A10	UNC13D
BMPR2	DHH	GUCY2F	MAMLD1	PGR	SLC30A2	UNC5C
BOC	DHRS4L1	GYG1	MAN1A2	PGRMC1	SLC30A5	UNC5CL
BPGM	DHTKD1	GYG2	MAN2A1	PHB	SLC30A8	UNC93A
BPI	DHX36	GYPA	MAOA	PHEX	SLC31A1	UNC93B1
BPIFA1	DIABLO	GYPB	MAOB	PHF11	SLC34A1	UNG
BRAP	DIAPH2	GYPC	MAP2	PHF2	SLC34A2	UNKL
BRCC3	DIAPH3	GYPE	MAP2K3	PHF3	SLC34A3	UPB1
BRD1	DIO1	GYS1	MAP2K4	PHF8	SLC35D1	UPF3B
BRD2	DIO2	GYS2	MAP2K7	PHKA1	SLC35G2	UPK3A
BRSK2	DIP2A	GZMB	MAP3K1	PHKA2	SLC44A2	UQCRB
BRWD1	DIP2B	H2BFWT	MAP3K14	PHKB	SLC46A1	UROD
BRWD3	DIP2C	H6PD	MAP3K15	PHKG2	SLC47A1	USP1
BSCL2	DIRC2	HABP2	MAP3K8	PHLPP2	SLC47A2	USP15
BSG	DISC1	HAL	MAP4K5	PI3	SLC4A1	USP24
BSND	DISP1	HAMP	MAP6	PICALM	SLC4A10	USP26
BST1	DKK2	HAND1	MAP7D3	PICK1	SLC4A3	USP46
BTAF1	DKK3	HAND2	MAPK10	PIF1	SLC4A7	USP9Y
BTBD9	DLC1	HAPLN1	MAPK8IP1	PIGM	SLC52A1	UST
BTC	DLEC1	HARS2	MARVELD2	PIGR	SLC5A1	UTF1

BTLA	DLG5	HAS1	MASP2	PIGZ	SLC5A11	UTP4
BTN1A1	DLGAP2	HAVCR1	MAST4	PIK3C2G	SLC5A2	UTRN
BTN2A1	DLGAP3	HAX1	MASTL	PIK3C3	SLC5A5	UTS2
BTRC	DLL3	HBD	MAT1A	PIK3CB	SLC6A1	UVSSA
C10orf105	DLX3	HBE1	MATN3	PIK3CG	SLC6A11	VANGL1
C10orf11	DLX6	HBEGF	MATR3	PIK3R4	SLC6A12	VAPB
C12orf29	DMBT1	HBG2	MAVS	PIM1	SLC6A13	VCAM1
C12orf65	DMC1	HBM	MBD1	PIN1	SLC6A14	VCL
C15orf62	DMD	HBS1L	MBD3	PIP4K2A	SLC6A18	VCX3A
C16orf58	DMGDH	HBZ	MBD4	PIP5K1B	SLC6A2	VDR
C1GALT1	DMP1	HCFC1	MBL2	PIP5K1C	SLC6A4	VEGFA
C1GALT1C1	DMRT1	HCK	MBNL1	PITPNA	SLC6A5	VIP
C1QA	DMXL1	HCLS1	MC2R	PIWIL3	SLC6A6	VIPAS39
C1QB	DNAJA4	HCN2	MC3R	PKD1	SLC7A1	VIPR2
C1QC	DNAJB2	HCN3	MC4R	PKD1L1	SLC7A10	VKORC1
C1S	DNAJB6	HCN4	MCC	PKD2	SLC7A11	VNN1
C21orf91	DNASE1L1	HCRTR1	MCCC1	PKHD1	SLC7A2	VPREB1
C2orf42	DNASE2	HCRTR2	MCCC2	PKLR	SLC7A5	VPS33B
C3	DNMT3L	HDAC9	MCEE	PKM	SLC7A7	VPS37A
C3AR1	DOC2A	HDC	MCF2L2	PKN3	SLC7A9	VPS54
C4BPA	DOCK4	HDLBP	MCFD2	PKP1	SLC8A1	VRK1
C5	DOCK9	HDX	MCHR1	PLA2G2A	SLC9A3R1	VSIG4
C5AR2	DOK1	HELQ	MCL1	PLA2G2D	SLC9A9	VTN
C6	DOK2	HEPACAM	MCM4	PLA2G4A	SLCO1A2	VWF
C7	DOK5	HEPH	MCM6	PLA2G4C	SLCO1B1	WASF3
C8A	DOLK	HES6	MCM7	PLA2G7	SLCO1B3	WDFY4
C8B	DOLPP1	HES7	MCM9	PLAG1	SLCO1C1	WDR13
C9	DOT1L	HEY1	MCPH1	PLAGL1	SLCO2B1	WDR3
CA1	DPCD	HEY2	MDH1	PLAT	SLCO5A1	WDR62
CA12	DPP10	HFE2	MDM2	PLAU	SLFN5	WDR72
CA6	DPY19L2	HGF	MDM4	PLAUR	SLIT3	WISP3
CABIN1	DPYS	HHEX	MDN1	PLCB1	SLITRK1	WNK4
CABP2	DPYSL2	HHIP	ME2	PLCE1	SLITRK5	WNT4
CACNA1C	DRD1	HIF1A	MECOM	PLCZ1	SLURP1	WNT5B
CACNA2D1	DRD2	HIF1AN	MED17	PLD2	SMAD1	WNT7A
CACNA2D3	DRD3	HINT1	MEF2A	PLEKHG4	SMAD2	WWC1
CACNB2	DRD4	HIP1	MEGF10	PLEKHG5	SMAD5	WWTR1
CACNG3	DRD5	HIST3H3	MEGF11	PLIN1	SMAD6	XBP1

CACNG4	DROSHA	HK2	MEIS1	PLIN4	SMAD7	XDH
CADM1	DRP2	HLX	MEP1B	PLOD2	SMAD9	XG
CADPS2	DSC3	HMBS	MEPE	PLSCR3	SMAP1	XIAP
CALCA	DSCAM	HMGA1	MESDC2	PLTP	SMARCA2	XIST
CALCR	DSCR8	HMGCL	MESP2	PLXND1	SMARCAD1	XK
CALCRL	DSG1	HMGCR	MEST	PMAIP1	SMC1B	XKR4
CALHM1	DSG3	HMGCS2	MET	PML	SMG1	XPNPEP2
CALM1	DSPP	HMMR	METTL21A	PMS2P3	SMG6	XRCC3
CALM3	DTNA	HMOX1	MFAP4	PNLIP	SMIM3	XRCC5
CALR3	DUOX2	HMOX2	MFGE8	PNP	SMN1	XRCC6
CALU	DUOXA1	HMSD	MFSD2A	PNPLA2	SMN2	YARS
CAMK4	DUSP23	HMX2	MGAT1	PNPLA3	SMNDC1	YBX2
CAMKK1	DYM	HNF1B	MGAT3	POF1B	SMOC2	YTHDF2
CAMKK2	DYNAP	HNRNPH3	MGAT4A	POFUT2	SMPD3	YWHAE
CAMKMT	DYSF	HOGA1	MGAT4B	POLB	SMPX	ZAN
CAMP	DYX1C1	HOMER2	MGAT4C	POLE2	SMUG1	ZAP70
CAMSAP2	E2F1	HOXA10	MGAT5	POLL	SMYD3	ZBTB25
CAPN10	E2F4	HOXA11	MGAT5B	POLR2E	SNAPC4	ZBTB40
CAPN13	E2F5	HOXA2	MGEA5	POLR2F	SNAPC5	ZBTB41
CAPN3	EBAG9	HOXA3	MGLL	POLR3H	SNCAIP	ZC3H3
CARD11	ECE1	HOXA4	MGMT	POLRMT	SNCB	ZC3HAV1
CARD14	ECE2	HOXB6	MGST2	POMC	SND1	ZCCHC12
CARD8	ECI1	HOXC13	MGST3	POMP	SNORD116-10	ZCCHC13
CARD9	ECM1	HOXD10	MIA3	PON1	SNORD50A	ZCCHC8
CARTPT	ECSIT	HOXD13	MIAT	PON2	SNRK	ZDHHC15
CASC16	EDA	HOXD4	MICAL1	PON3	SNTA1	ZDHHC17
CASP1	EDA2R	HP	MIIP	POP1	SNTG2	ZDHHC24
CASP12	EDAR	HPD	MINPP1	POSTN	SNX19	ZDHHC6
CASP2	EDARADD	HPRT1	MIPOL1	POU4F3	SNX3	ZDHHC8
CASP3	EDN2	HPSE2	MIR146A	POU5F1	SOCS1	ZFAT
CASP5	EEF1B2	HR	MIR17HG	POU5F1B	SOCS3	ZFHX3
CASP8	EEF2K	HRC	MIR206	PPARA	SOD1	ZFP36
CASP9	EEF2KMT	HRG	MIR510	PPARD	SOD2	ZFP36L1
CAST	EFCAB5	HRH2	MIR96	PPARG	SOD3	ZFP36L2
CAT	EFHC2	HRH3	MKL1	PPARGC1A	SOGA3	ZFP69
CATSPER1	EFNA5	HS1BP3	MLC1	PPARGC1B	SOHLH1	ZFP90
CATSPER2	EFR3A	HSD11B1	MLLT10	PPAT	SORBS1	ZFYVE27
CATSPER3	EFTUD2	HSD17B1	MLLT3	PPIA	SORCS1	ZHX3

CATSPER4	EGF	HSD17B2	MLYCD	PPIG	SORL1	ZIC4
CAV3	EGLN1	HSD17B3	MMAA	PPM1B	SORT1	ZMYM3
CBFB	EGR3	HSD3B1	MMAB	PPM1G	SOX17	ZNF175
CBLB	EHBP1	HSD3B2	MMEL1	PPM1K	SOX18	ZNF202
CBR1	EHD2	HSF1	MMP10	PPOX	SOX7	ZNF213
CBR3	EHHADH	HSP90AA1	MMP12	PPP1R1A	SP100	ZNF224
CBX2	EIF3H	HSP90B1	MMP13	PPP1R3A	SP110	ZNF24
CBX4	EIF4E	HSPA5	MMP20	PPP1R3C	SP7	ZNF300
CBY1	EIF4H	HSPA8	MMP3	PPP2R1B	SP8	ZNF350
CCDC107	ELAC2	HSPA9	MMP7	PPP2R2C	SPAG16	ZNF385B
CCDC12	ELANE	HSPB1	MMP8	PPP3R1	SPAG17	ZNF41
CCDC127	ELAVL2	HSPB3	MMP9	PRB1	SPAG8	ZNF419
CCDC14	ELF4	HSPB7	MNX1	PRB3	SPANXN5	ZNF420
CCDC170	ELK1	HSPB8	MOCOS	PRB4	SPATA13	ZNF433
CCDC50	ELK3	HTN3	MOK	PRCC	SPATA16	ZNF480
CCDC66	ELMOD2	HTR1A	MPG	PRCP	SPATA21	ZNF507
CCDC78	ELP2	HTR1B	MPHOSPH8	PRDM2	SPATA31C1	ZNF526
CCDC8	EME1	HTR2A	MPI	PRDM9	SPECC1	ZNF592
CCK	EMG1	HTR2B	MPO	PRG4	SPG20	ZNF627
CCKAR	EMX1	HTR2C	MPP3	PRH1	SPG21	ZNF674
CCKBR	EMX2	HTR3A	MPP4	PRICKLE1	SPI1	ZNF711
CCL11	EN2	HTR3B	MPP6	PRICKLE2	SPINK1	ZNF75D
CCL17	ENAM	HTR3C	MPP7	PRKAA2	SPINK5	ZNF80
CCL2	ENO1	HTR3E	MPST	PRKACA	SPP1	ZNF804A
CCL22	ENO3	HTR5A	MR1	PRKAG3	SPRED2	ZNF81
CCL26	ENSA	HTR6	MRAP	PRKAR1B	SPRN	ZNHIT6
CCL3	ENTPD5	HTR7	MRC1	PRKCA	SPRR3	ZNRF1
CCL5	EOMES	HVCN1	MREG	PRKCB	SPRY2	ZPBP
CCL7	EPB41	IAPP	MRPL3	PRKCH	SPTA1	ZPBP2
CCNA2	EPB42	IBSP	MRPL48	PRKCSH	SPTAN1	
CCNH	EPC2	ICAM1	MRPS22	PRKD3	SPTB	
CCPG1	EPHA3	ICAM4	MRRF	PRKRA	SPTBN1	
CCR1	EPHA5	ICAM5	MS4A1	PRL	SPTBN5	
CCR2	EPHA7	ICK	MS4A12	PRLH	SPTLC1	
CCR3	EPHB2	ID3	MS4A2	PRLHR	SPTLC2	
CCR5	EPHB6	ID4	MS4A3	PRLR	SRD5A2	
CCR6	EPHX1	IDE	MS4A6A	PRM1	SREBF2	
CCR7	EPO	IDH1	MS4A6E	PRM2	SREK1	

Table S11. List with 100 VUS pending on reclassification at the Genetics Department of the HU-FJD.

ID	HGVSc	HGVSp	SYMBOL	AC	AF	AC_IRD	AF_IRD	AC_PC	AF_PC
chr1:94471103A>G	NM_000350.3: c.6041T>C	NP_000341.2: p.Met2014Thr	ABCA4	1	8.80E-05	1	2.90E-04	0	0.00E+00
chr1:94502731A>C	NM_000350.3: c.3783T>G	NP_000341.2: p.Ser1261Arg	ABCA4	1	8.97E-05	1	2.90E-04	0	0.00E+00
chr1:94506911G>C	NM_000350.3: c.3376C>G	NP_000341.2: p.Leu1126Val	ABCA4	1	8.77E-05	1	2.80E-04	0	0.00E+00
chr1:94526199G>A	NM_000350.3: c.2054C>T	NP_000341.2: p.Thr685Ile	ABCA4	1	8.80E-05	1	2.80E-04	0	0.00E+00
chr1:103352419G>T	NM_001190709.1: c.4685C>A	NP_001177638.1: p.Thr1562Asn	COL11A1	6	5.33E-04	3	8.60E-04	2	2.86E-04
chr1:103474020T>C	NM_001190709.1: c.1565A>G	NP_001177638.1: p.Gln522Arg	COL11A1	1	8.98E-05	1	2.90E-04	0	0.00E+00
chr1:150316692C>T	NM_001350529.1: c.1076C>T	NP_001337458.1: p.Thr359Met	PRPF3	2	1.81E-04	2	5.90E-04	0	0.00E+00
chr1:186120331G>A	NM_031935.3: c.14609-1G>A	NA	HMCN1	1	8.97E-05	1	2.90E-04	0	0.00E+00
chr1:197297911T>G	NM_001193640.2: c.430T>G	NP_001180569.1: p.Phe144Val	CRB1	14	1.26E-03	7	2.05E-03	5	7.26E-04
chr1:197297973GGATGGAATT>G	NM_001193640.2: c.498_506del	NP_001180569.1: p.Ile167_Gly169del	CRB1	37	3.25E-03	22	6.24E-03	14	1.99E-03
chr1:197298095T>C	NM_001193640.2: c.614T>C	NP_001180569.1: p.Ile205Thr	CRB1	38	3.33E-03	15	4.25E-03	23	3.26E-03
chr1:202910771T>C	NM_001290553.1: c.1058A>G	NP_001277482.1: p.Tyr353Cys	ADIPOR1	1	9.03E-05	1	2.90E-04	0	0.00E+00
chr1:215799146T>G	NA	NA	KCTD3	1	9.03E-05	1	2.90E-04	0	0.00E+00
chr1:215848154C>T	NM_206933.3: c.13099G>A	NP_996816.2: p.Val4367Ile	USH2A	1	8.77E-05	1	2.80E-04	0	0.00E+00
chr1:215848921G>A	NM_206933.3: c.12332C>T	NP_996816.2: p.Ser4111Phe	USH2A	1	8.77E-05	1	2.80E-04	0	0.00E+00
chr1:215960035C>A	NM_206933.3: c.10364G>T	NP_996816.2: p.Ser3455Ile	USH2A	1	8.77E-05	1	2.80E-04	0	0.00E+00
chr1:216246603C>T	NM_206933.3: c.5612G>A	NP_996816.2: p.Gly1871Asp	USH2A	9	7.90E-04	2	5.70E-04	6	8.50E-04
chr1:216256830C>T	NM_206933.3: c.5266G>A	NP_996816.2: p.Val1756Ile	USH2A	1	8.82E-05	1	2.90E-04	0	0.00E+00
chr1:216258156G>A	NM_206933.3: c.5051C>T	NP_996816.2: p.Pro1684Leu	USH2A	1	8.77E-05	1	2.80E-04	0	0.00E+00
chr1:216500940T>G	NM_007123.5: c.841A>C	NP_009054.5: p.Thr281Pro	USH2A	2	1.77E-04	1	2.90E-04	1	1.43E-04
chr10:73498276G>A	NA	NA	C10orf105	3	2.63E-04	2	5.70E-04	1	1.42E-04
chr10:85961599G>A	NM_001171971.3: c.562G>A	NP_001165442.1: p.Gly188Ser	CDHR1	1	8.88E-05	1	2.90E-04	0	0.00E+00
chr10:85971932C>A	NM_001171971.3: c.1554-3C>A	NA	CDHR1	5	4.40E-04	2	5.70E-04	3	4.26E-04
chr10:85971970C>G	NM_001171971.3: c.1589C>G	NP_001165442.1: p.Thr530Ser	CDHR1	4	3.57E-04	3	8.70E-04	1	1.44E-04
chr10:102780410T>C	NM_001195263.2: c.893A>G	NP_001182192.1: p.Lys298Arg	PDZD7	1	8.93E-05	1	2.90E-04	0	0.00E+00
chr11:61725731C>G	NM_001139443.2: c.648C>G	NP_001132915.1: p.Phe216Leu	BEST1	1	8.84E-05	1	2.90E-04	0	0.00E+00
chr12:88448181G>A	NA	NA	C12orf29	1	8.98E-05	1	2.90E-04	0	0.00E+00

chr12:88481670G>C	NM_025114.4: c.4081C>G	NP_079390.3: p.Leu1361Val	CEP290	1	8.85E-05	1	2.90E-04	0	0.00E+00
chr12:88535042G>T	NA	NA	TMTC3	1	8.89E-05	1	2.90E-04	0	0.00E+00
chr14:68196055C>G	NM_152443.3: c.806C>G	NP_689656.2: p.Ala269Gly	RDH12	1	8.79E-05	1	2.80E-04	0	0.00E+00
chr14:88892973G>A	NM_001040428.3: c.674G>A	NP_001035518.1: p.Arg225His	SPATA7	3	2.69E-04	1	2.90E-04	2	2.88E-04
chr16:57921818G>A	NM_001286130.2: c.3385C>T	NP_001273059.1: p.Arg1129Trp	CNGB1	1	8.90E-05	1	2.90E-04	0	0.00E+00
chr16:57973358C>G	NM_001286130.2: c.1330G>C	NP_001273059.1: p.Glu444Gln	CNGB1	1	9.66E-05	1	3.30E-04	0	0.00E+00
chr17:1554979T>G	NM_006445.4: c.6473A>C	NP_006436.3: p.His2158Pro	PRPF8	1	8.87E-05	1	2.90E-04	0	0.00E+00
chr17:1558750T>C	NM_006445.4: c.5881A>G	NP_006436.3 :p.Ile1961Val	PRPF8	1	9.02E-05	1	2.90E-04	0	0.00E+00
chr17:6328998C>A	NM_001033054.3: c.748G>T	NP_001028226.1: p.Ala250Ser	AIPL1	18	1.62E-03	6	1.75E-03	12	1.74E-03
chr17:6337375G>C	NM_001033054.3: c.140C>G	NP_001028226.1: p.Thr47Arg	AIPL1	17	1.49E-03	6	1.70E-03	11	1.56E-03
chr17:7906552G>A	NM_000180.4: c.187G>A	NP_000171.1: p.Ala63Thr	GUCY2D	1	8.89E-05	1	2.90E-04	0	0.00E+00
chr17:7917341G>C	NM_000180.4: c.2407G>C	NP_000171.1: p.Asp803His	GUCY2D	1	9.00E-05	1	2.90E-04	0	0.00E+00
chr17:7918305T>C	NM_000180.4: c.2705T>C	NP_000171.1: p.Val902Ala	GUCY2D	1	8.77E-05	1	2.80E-04	0	0.00E+00
chr17:26879514G>A	NM_001330166.2: c.-252C>T	NA	UNC119	1	9.04E-05	1	2.90E-04	0	0.00E+00
chr17:58234860T>C	NM_000717.5: c.341T>C	NP_000708.1: p.Leu114Ser	CA4	10	9.01E-04	3	8.80E-04	6	8.70E-04
chr17:79496011G>A	NM_001077182.3: c.454G>A	NP_001070650.1: p.Val152Met	FSCN2	1	8.77E-05	0	0.00E+00	1	1.42E-04
chr17:79503252TGAA>T	NM_001077182.3: c.1071_1073del	NP_001070650.1: p.Lys357del	FSCN2	2	1.75E-04	0	0.00E+00	0	0.00E+00
chr17:79503273C>T	NM_001077182.3: c.1085C>T	NP_001070650.1: p.Ala362Val	FSCN2	1	8.77E-05	1	2.80E-04	0	0.00E+00
chr19:3770708C>T	NM_001319074.2: c.604G>A	NP_001306003.1: p.Ala202Thr	RAX2	1	8.93E-05	1	2.90E-04	0	0.00E+00
chr19:7621417C>T	NM_001166111.2: c.3202C>T	NP_001159583.1: p.Arg1068Cys	PNPLA6	2	1.75E-04	1	2.80E-04	1	1.42E-04
chr19:54628040G>A	NM_015629.4: c.855+5G>A	NA	PRPF31	1	8.82E-05	1	2.80E-04	0	0.00E+00
chr2:29296025TGCTTGCCCA>T	NM_001029883.3: c.1094_1102del	NP_001025054.1: p.Leu365_Lys367del	C2orf71	2	1.75E-04	2	5.70E-04	0	0.00E+00
chr2:98986517C>T	NM_001079878.2: c.79C>T	NP_001073347.1: p.Arg27Cys	CNGA3	1	8.88E-05	1	2.90E-04	0	0.00E+00
chr2:99012444C>G	NM_001079878.2: c.757C>G	NP_001073347.1: p.Pro253Ala	CNGA3	15	1.32E-03	5	1.42E-03	7	9.93E-04
chr2:99012834T>C	NM_001079878.2: c.1147T>C	NP_001073347.1: p.Ser383Pro	CNGA3	2	1.75E-04	2	5.70E-04	0	0.00E+00
chr2:99013422G>A	NM_001079878.2: c.1735G>A	NP_001073347.1: p.Ala579Thr	CNGA3	2	1.75E-04	1	2.80E-04	1	1.42E-04
chr2:112740548T>A	NM_006343.3: c.1274T>A	NP_006334.2: p.Val425Glu	MERTK	1	8.78E-05	1	2.80E-04	0	0.00E+00
chr2:112779018G>A	NM_006343.3: c.2209G>A	NP_006334.2: p.Val737Ile	MERTK	1	8.77E-05	1	2.80E-04	0	0.00E+00
chr2:112779920A>G	NM_006343.3: c.2435A>G	NP_006334.2: p.Tyr812Cys	MERTK	3	2.63E-04	2	5.70E-04	1	1.42E-04

chr2:182409512C>T	NM_001030311.2: c.1358G>A	NP_001025482.1: p.Gly453Glu	CERKL	1	8.87E-05	0	0.00E+00	1	1.43E-04
chr2:234217866G>A	NM_000541.5: c.31G>A	NP_000532.2: p.Glu11Lys	SAG	20	1.80E-03	4	1.17E-03	14	2.03E-03
chr20:3891453A>T	NM_001324191.2: c.338A>T	NP_001311120.1: p.Asn113Ile	PANK2	4	3.60E-04	3	8.70E-04	1	1.45E-04
chr3:50231006C>A	NM_000172.4: c.359C>A	NP_000163.2: p.Ser120Ter	GNAT1	7	6.14E-04	2	5.70E-04	5	7.08E-04
chr3:100962441ACAT>A	NM_016247.4: c.2731_2733del	NP_057331.2: p.Met911del	IMPG2	1	9.32E-05	1	3.00E-04	0	0.00E+00
chr3:100964729T>A	NM_016247.4: c.1460A>T	NP_057331.2: p.His487Leu	IMPG2	2	1.75E-04	2	5.70E-04	0	0.00E+00
chr3:100964889G>A	NM_016247.4: c.1300C>T	NP_057331.2: p.Pro434Ser	IMPG2	11	9.74E-04	5	1.43E-03	5	7.16E-04
chr3:193366624A>T	NM_001354663.2: c.1442A>T	NP_001341592.1: p.Glu481Val	OPA1	1	9.03E-05	1	2.90E-04	0	0.00E+00
chr4:619687G>T	NM_000283.3: c.272G>T	NP_000274.2: p.Arg91Leu	PDE6B	1	8.89E-05	0	0.00E+00	0	0.00E+00
chr4:6292935G>A	NM_001145853.1: c.472G>A	NP_001139325.1: p.Glu158Lys	WFS1	1	8.82E-05	1	2.80E-04	0	0.00E+00
chr4:6303119C>T	NM_001145853.1: c.1597C>T	NP_001139325.1: p.Pro533Ser	WFS1	6	5.26E-04	4	1.13E-03	0	0.00E+00
chr4:16026888C>T	NM_001145847.2: c.530G>A	NP_001139319.1: p.Arg177Gln	PROM1	1	8.97E-05	1	2.90E-04	0	0.00E+00
chr6:42141500C>T	NM_000409.4: c.149C>T	NP_000400.2: p.Pro50Leu	GUCA1A	37	3.30E-03	10	2.90E-03	24	3.45E-03
chr6:42147099A>AGACGAGGAGGGGCT	NM_000409.4: c.572_586dup	NP_000400.2: p.Glu191_Glu195dup	GUCA1A	1	9.03E-05	1	2.90E-04	0	0.00E+00
chr6:42672150G>A	NM_000322.5: c.781C>T	NP_000313.2: p.Leu261Phe	PRPH2	1	8.77E-05	1	2.80E-04	0	0.00E+00
chr6:42672265GCAGGGC>G	NM_000322.5: c.660_665del	NP_000313.2: p.Pro221_Cys222del	PRPH2	2	1.75E-04	2	5.70E-04	0	0.00E+00
chr6:42689961C>T	NM_000322.5: c.112G>A	NP_000313.2: p.Gly38Arg	PRPH2	1	8.77E-05	1	2.80E-04	0	0.00E+00
chr6:65303156G>A	NM_001142800.2: c.3731C>T	NP_001136272.1: p.Thr1244Ile	EYS	2	1.75E-04	2	5.70E-04	0	0.00E+00
chr6:66205150C>G	NM_001142800.2: c.154G>C	NP_001136272.1: p.Asp52His	EYS	1	8.93E-05	1	2.90E-04	0	0.00E+00
chr6:66205279G>T	NM_001142800.2: c.25C>A	NP_001136272.1: p.Leu9Met	EYS	1	8.93E-05	1	2.90E-04	0	0.00E+00
chr6:70990715C>T	NM_001851.5: c.904G>A	NP_001842.3: p.Gly302Ser	COL9A1	6	5.41E-04	2	5.80E-04	4	5.81E-04
chr7:23145649G>A	NM_001031710.3: c.4G>A	NP_001026880.2: p.Ala2Thr	KLHL7	1	8.77E-05	1	2.80E-04	0	0.00E+00
chr7:128035018C>T	NM_000883.4: c.1475G>A	NP_000874.2: p.Arg492Gln	IMPDH1	6	5.41E-04	6	1.75E-03	0	0.00E+00
chr7:128041130G>A	NM_000883.4: c.443C>T	NP_000874.2: p.Thr148Met	IMPDH1	2	1.78E-04	1	2.90E-04	1	1.43E-04
chr8:10466978G>A	NM_178857.6: c.4630C>T	NP_849188.4: p.Arg1544Cys	RP1L1	1	1.05E-04	1	3.50E-04	0	0.00E+00
chr8:10469047G>A	NM_178857.6: c.2561C>T	NP_849188.4: p.Pro854Leu	RP1L1	1	1.05E-04	1	3.50E-04	0	0.00E+00
chr8:10480383G>C	NM_178857.6: c.329C>G	NP_849188.4: p.Pro110Arg	RP1L1	1	8.78E-05	0	0.00E+00	1	1.42E-04
chr8:10480420C>T	NM_178857.6: c.292G>A	NP_849188.4: p.Asp98Asn	RP1L1	6	5.33E-04	3	8.60E-04	3	4.30E-04

chr8:43025820C>T	NM_001363227.2: c.726C>T	NP_001350156.1: p.Ser242%3D	HGSNAT	2	1.77E-04	1	2.90E-04	1	1.43E-04
chr8:43046725C>T	NM_001363227.2: c.1237C>T	NP_001350156.1: p.Pro413Ser	HGSNAT	7	6.14E-04	1	2.80E-04	6	8.50E-04
chr8:55533586A>C	NM_006269.2: c.60A>C	NP_006260.1: p.Gln20His	RP1	1	8.80E-05	1	2.80E-04	0	0.00E+00
chr8:55534144G>A	NM_006269.2: c.615+3G>A	NA	RP1	13	1.14E-03	5	1.42E-03	8	1.14E-03
chr8:55538939T>C	NM_006269.2: c.2497T>C	NP_006260.1: p.Phe833Leu	RP1	2	1.80E-04	1	2.90E-04	0	0.00E+00
chr8:87638255T>C	NM_019098.4: c.1534A>G	NP_061971.3: p.Ile512Val	CNGB3	12	1.06E-03	2	5.70E-04	9	1.28E-03
chr8:97172796C>A	NM_001001557.4: c.125G>T	NP_001001557.1: p.Gly42Val	GDF6	3	2.63E-04	2	5.70E-04	1	1.42E-04
chr9:2718887G>T	NM_133497.4: c.1148G>T	NP_598004.1: p.Arg383Leu	KCNV2	3	2.63E-04	1	2.80E-04	2	2.83E-04
chrX:13753441T>G	NA	NA	TRAPPC2	2	1.77E-04	2	5.80E-04	0	0.00E+00
chrX:18674836C>T	NM_000330.4: c.121G>A	NP_000321.1: p.Asp41Asn	RS1	2	1.75E-04	2	5.70E-04	0	0.00E+00
chrX:38178172T>C	NM_000328.3: c.379A>G	NP_000319.1: p.Arg127Gly	RPGR	1	9.02E-05	1	2.90E-04	0	0.00E+00
chrX:41333211A>G	NM_022567.2: c.505A>G	NP_072089.1: p.Asn169Asp	NYX	2	1.94E-04	2	6.30E-04	0	0.00E+00
chrX:41333709C>T	NM_022567.2: c.1003C>T	NP_072089.1: p.Arg335Cys	NYX	1	8.89E-05	1	2.90E-04	0	0.00E+00
chrX:49062162G>A	NM_001256789.3: c.5584C>T	NP_001243718.1: p.Arg1862Cys	CACNA1F	2	1.76E-04	1	2.80E-04	1	1.42E-04
chrX:49067552T>A	NM_001256789.3: c.4261A>T	NP_001243718.1: p.Ile1421Phe	CACNA1F	2	1.79E-04	2	5.80E-04	0	0.00E+00
chrX:49068452G>C	NM_001256789.3: c.4009-3C>G	NA	CACNA1F	2	1.76E-04	2	5.70E-04	0	0.00E+00

Table S12. Carrier frequency (in pseudocontrol cases) and frequency in cases with inherited retinal dystrophies for the top 10 genes with higher carrier frequency.

Gene	Allele Count	Frequency	Frequency type
ABCA4	252	7,14	Carrier
USH2A	89	2,52	Carrier
PDE6A	36	1,02	Carrier
CEP290	32	0,91	Carrier
ADGRV1	27	0,76	Carrier
CNGB3	22	0,62	Carrier
EYS	22	0,62	Carrier
CRB1	20	0,57	Carrier
RP1L1	20	0,57	Carrier
NMNAT1	19	0,54	Carrier
ABCA4	375	21,23	IRD
USH2A	264	14,95	IRD
PDE6A	36	2,04	IRD
CEP290	33	1,87	IRD
ADGRV1	36	2,04	IRD
CNGB3	63	3,57	IRD
EYS	53	3,00	IRD
CRB1	49	2,77	IRD
RP1L1	30	1,70	IRD
NMNAT1	12	0,68	IRD

Supplementary Figures

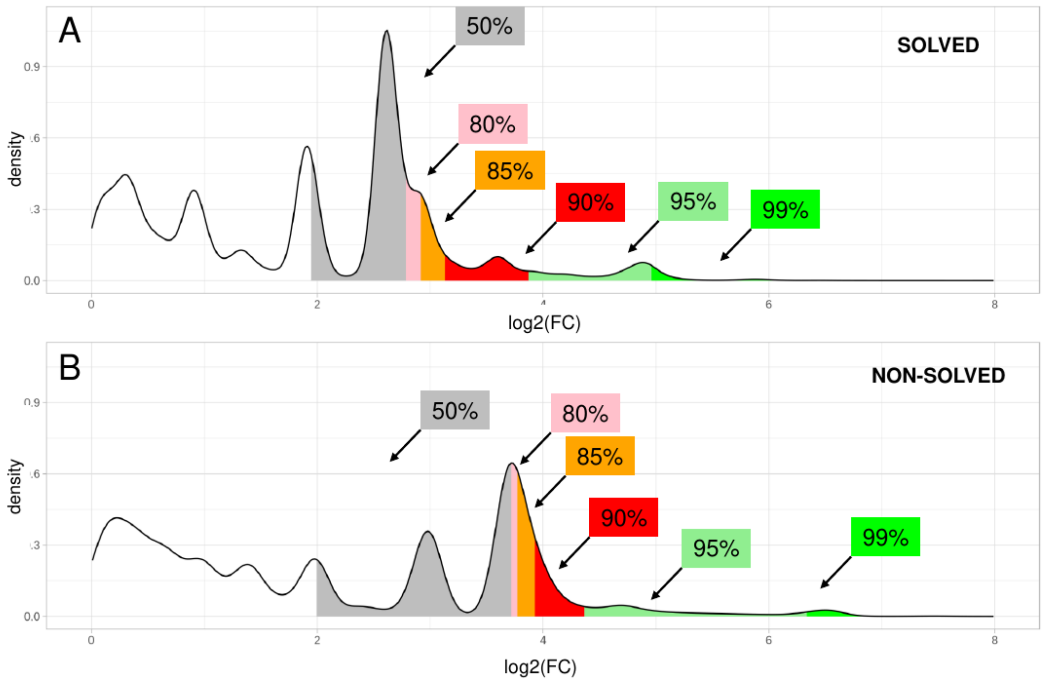

Figure S1. Distribution of the values of the fold changes (log2(FC)) calculated between the allelic frequencies in two IRD subcohorts: (A) solved and (B) non-solved, and the allelic frequencies in the pseudocontrols. Percentiles 50%, 80%, 85%, 90%, 95% and 99% are shown in both groups.

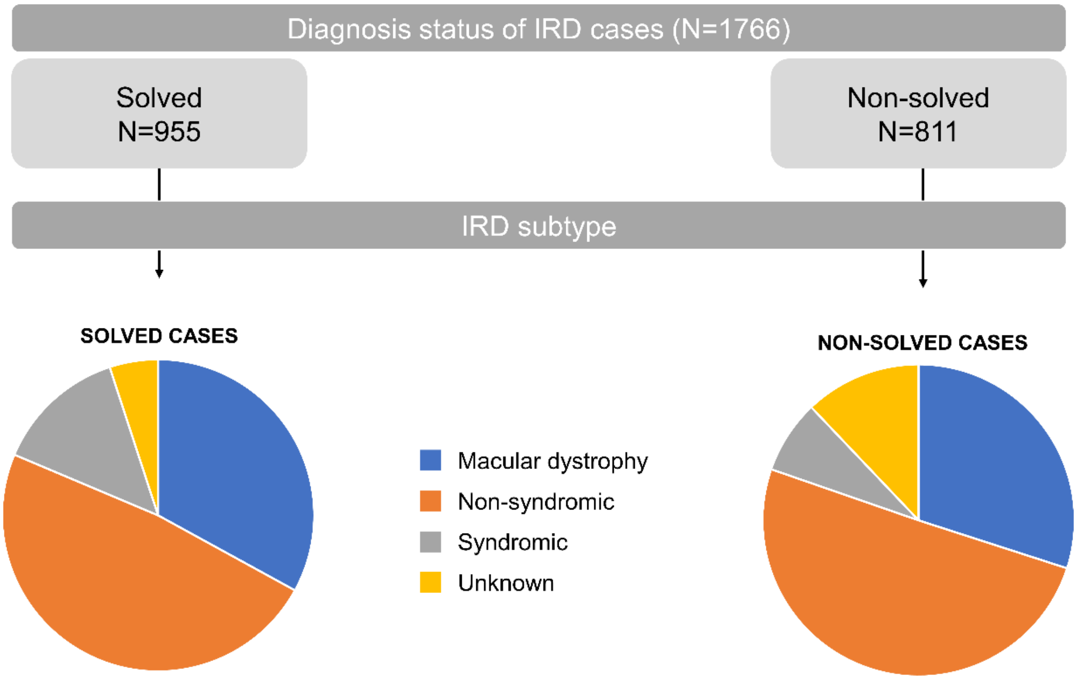

Figure S2. Description of the cohort of cases with inherited retinal dystrophies (IRD). Number of cases grouped by diagnostic status (solved and non-solved) and IRD subtype (syndromic, non-syndromic, macular dystrophies and unknown).

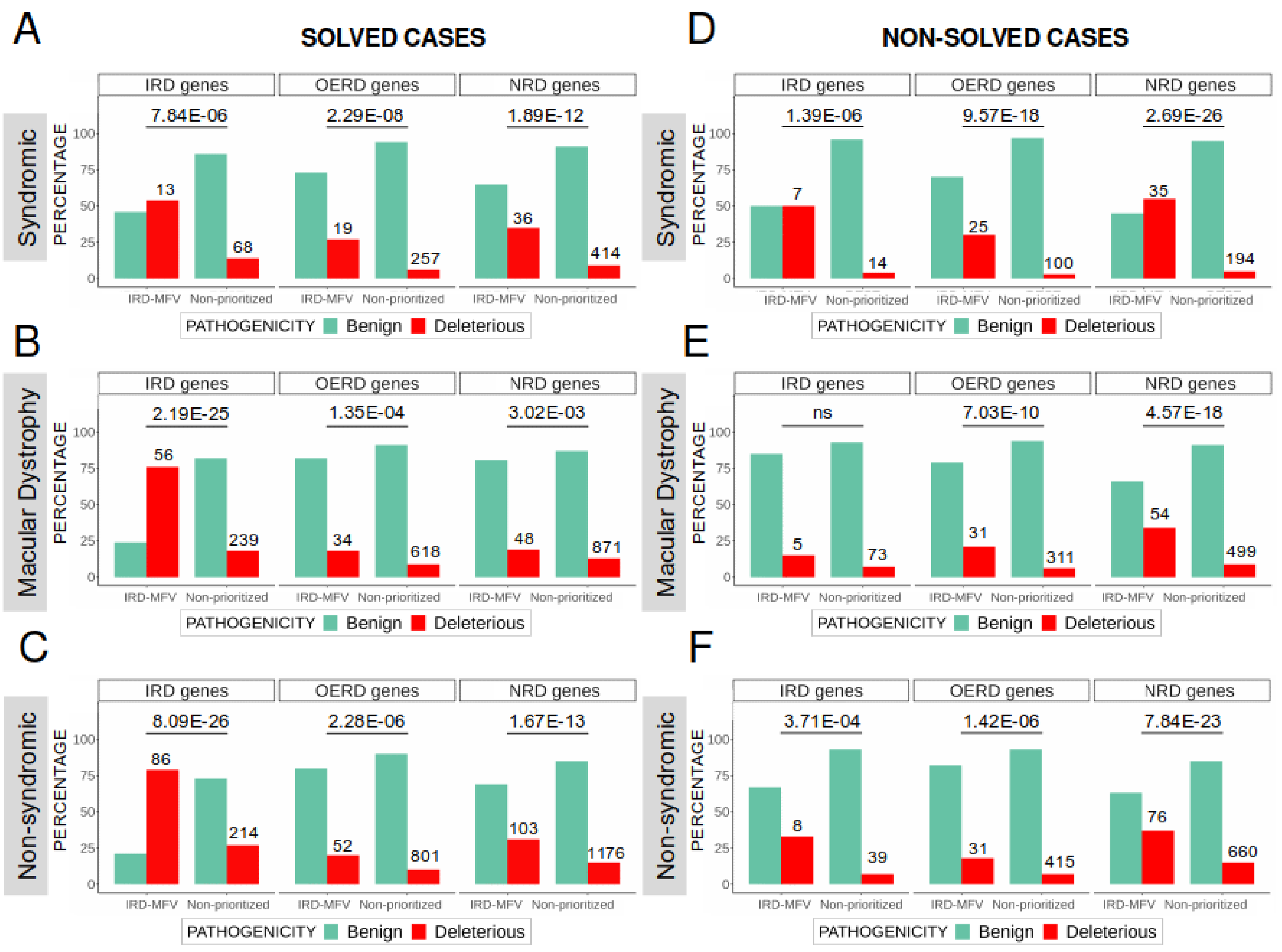

Figure S3. Proportion of deleterious and benign variants in both solved (A, B and C) and non-solved cases with inherited retinal dystrophies (D, E and F) for the different IRD subtypes: syndromic, non-syndromic and macular dystrophies. The p-values representing the enrichment of deleterious variants in IRD-MFVs are shown. The genes in which the IRD-MFVs are located are grouped in: inherited retinal dystrophies (RD genes), other eye related diseases (OERD genes) and other non-related diseases (NRD genes). Non-significant p-values are marked as “ns”.

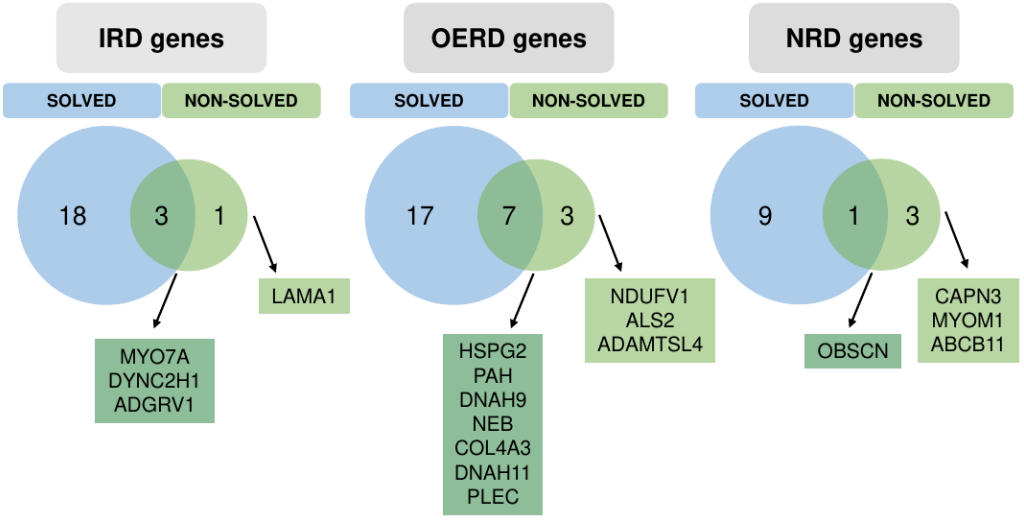

Figure S4. Intersection of genes prioritized in solved and non-solved cases with inherited retinal dystrophies. The genes are grouped as involved in: inherited retinal dystrophies (IRD genes), other eye related diseases (OERD genes) and other non-related diseases (NRD genes). We show only the names of the genes prioritized in non-solved IRD cases, in light green those unique to non-solved, and in dark green those in common with IRD solved cases.

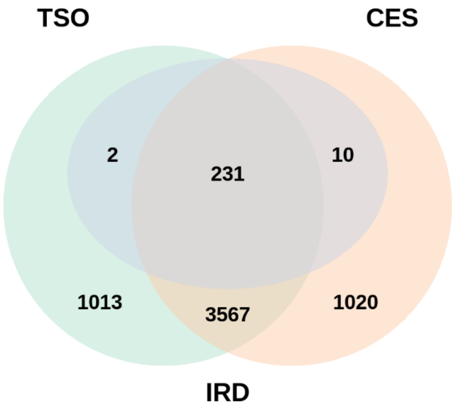

Figure S5. Intersection in genes included in the two clinical exomes used in the sequencing of the samples in the cohort: TruSightOne Sequencing Panel kit (TSO, Illumina, San Diego, CA), and Clinical Exome Solution Sequencing Panel kit (CES, Sophia Genetics, Boston, MA). Genes involved in inherited retinal dystrophies (IRD) are also highlighted.

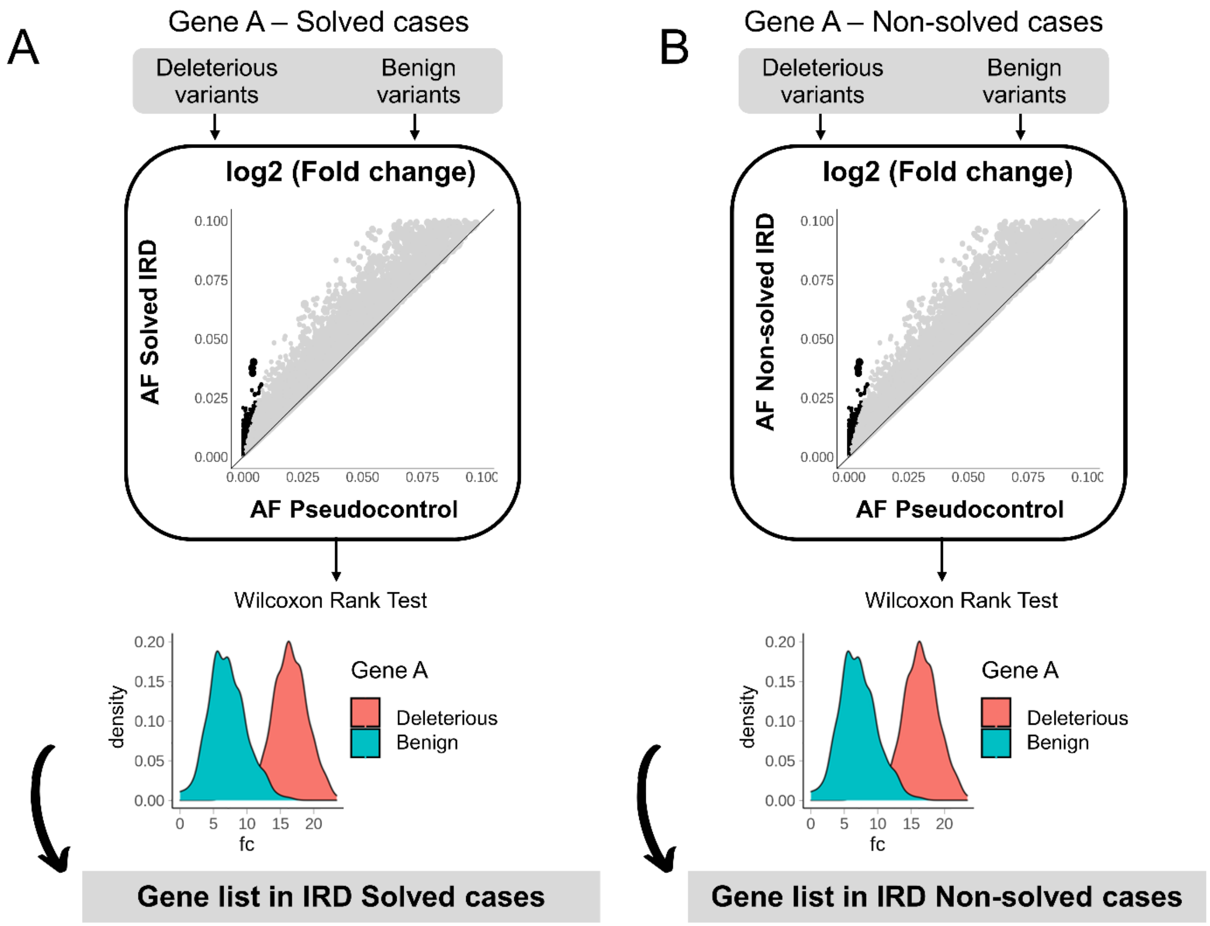

Figure S6. Workflow to perform gene prioritization in (A) solved and (B) non-solved cases with inherited retinal dystrophies (IRD).
